# Cancer‐Cell‐Specific Drug Delivery by a Tumor‐Homing CPP‐Gossypol Conjugate Employing a Tracelessly Cleavable Linker

**DOI:** 10.1002/chem.201905159

**Published:** 2020-01-30

**Authors:** Suman Kumar Maity, Paul Stahl, Astrid Hensel, Shirley Knauer, Christoph Hirschhäuser, Carsten Schmuck

**Affiliations:** ^1^ Institute of Organic Chemistry University of Duisburg-Essen Universitatsstrasse 7 45117 Essen Germany; ^2^ Institute for Biology University of Duisburg-Essen 45117 Essen Germany

**Keywords:** cleavable linker, drug delivery, gossypol, imine linkage, tumor-homing peptide

## Abstract

Tumor‐targeted drug delivery is highly important for improving chemotherapy, as it reduces the dose of cytotoxic agents and minimizes the death of healthy tissues. Towards this goal, a conjugate was synthesized of gossypol and a MCF‐7 cancer cell specific CPP (cell penetrating peptide), thus providing a selective drug delivery system. Utilizing the aldehyde moiety of gossypol, the tumor homing CPP RLYMRYYSPTTRRYG was attached through a semi‐labile imine linker, which was cleaved in a traceless fashion under aqueous conditions and had a half‐life of approximately 10 hours. The conjugate killed MCF‐7 cells to a significantly greater extent than HeLa cells or healthy fibroblasts.

## Graphical abstract

**Selective drug delivery**: A Cancer‐cell specific drug delivery system was developed by conjugating a tumor homing peptide and gossypol through a cleavable linker. The Schiff's base linked conjugate killed the targeted MCF‐7 cells selectively compared to HeLa cells used as a control (see figure).

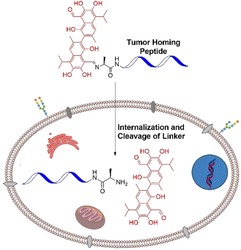

To date, cancer is one of the leading causes of global death due to the difficulties associated with tumor selective therapy, such as inefficient drug accumulation, cancer cell heterogeneity, and drug resistance.^1^ The nonspecific toxicity of anticancer agents towards healthy tissues is a major challenge in conventional chemotherapeutic treatments.^2^ Thus, targeted drug delivery, envisioned by Paul Ehrlich as a “magic bullet”, is a long standing research objective.^3^ Towards this goal, significant advancements were achieved by exploiting the advantages of different cancer specific vectors,^4^ such as antibodies,^5^ aptamers,^6^ folic acid derivatives^4^, ^7^ and cell penetrating peptides.^8^ Tumor‐homing peptides, which are small oligopeptides (3 to 15 residues) identified through sophisticated techniques (phage display, mRNA display), are evolving as specific vectors for cancer cells.^9^ The design of such targeted drug delivery systems relies majorly on the conjugation of anticancer drugs to the vectors via a cleavable linker. Synthetic organic chemistry aims towards developing suitably cleavable linkers, which release unmodified drugs over a multi‐hour timeframe under physiological conditions.^10^ Compared to antibodies and aptamers, tumor homing peptides are relatively easy to modify chemically and can be conjugated to drugs through cleavable linkers. Therefore, conjugating novel anticancer agents to tumor homing peptides in order to understand and harness their therapeutic potential is of major interest. Gossypol (AT101), an aldehyde containing phenol derived from the cotton plant, was initially explored as a male antifertility drug. It exhibited promising anticancer activities^11^ towards various tumors through different mechanisms including proliferation inhibition and apoptosis induction.^12^, ^13^ Its antiproliferative effect is caused by regulating cycline D1,^14^ autophagy,^15^ and inhibition of aldehyde dehydrogenase.^16^ Furthermore Gossypol is studied for combination therapy in addition with other therapeutic agents against glioblastoma,^17^ pancreatic cancer cells^18^ and non‐small‐cell lung carcinoma.^19^ It has also shown cytotoxicity against breast cancer cells by inhibiting the expression of mouse double minute 2 (MDM2) and vascular endothelial growth factor (VEGF).^20^ Currently gossypol is in phase II clinical trials as an anticancer drug. However, gossypol, as many other conventional anticancer drugs, faces a number of obstacles including bad water solubility, poor cellular uptake and a lack of selectivity. Therefore, reversible attachment of gossypol to a vector that enables cell membrane penetration, increases solubility and allows for addressing cancer cells selectively, seemed highly advantageous.

In this report, we describe the synthesis of cancer cell line specific peptide–gossypol conjugates and their cytotoxic effects. The aldehyde group of gossypol was utilized for conjugation, and thiazolidine (**1 a**) as well as imine linkages (**1 b**) were explored as traceless cleavable linkers (Figure 1). The cancer cell line specific cell penetrating peptide (CPP), RLYMRYYSPTTRRYG was developed by Matsushita and co‐wokers.9a It is specific to MCF‐7 breast cancer cells and was chosen as gossypol also showed anticancer activity on this cell line. This CPP is internalized into cells through a dynamin‐dependent endocytic pathway. Conjugation of this CPP to gossypol increased solubility of the hydrophobic drug (in working buffers: Dulbecco's Modified Eagle's Medium (DMEM) and RPMI medium supplemented with 10 % FBS, streptomycin sulfate (0.1 mg mL^−1^), penicillin (10 U mL^−1^) and amphotericin B (0.25 μg mL^−1^), at pH 7.4); this makes the use of potentially harmful solubilizing agents superfluous. Initial attempts to synthesize the thiazolidine linked conjugate **1 a** were based on standard procedures from the literature,^24^ which led to the formation of byproducts among which also was imine **1 b**. The inseparable mixture of conjugates **1 a** and **1 b** obtained from the above reaction exhibited specific toxicity to MCF‐7 cells compared to HeLa cells. Time‐dependent cytotoxicity assays revealed that imine **1 b** was much more efficient in killing MCF‐7 cells compared to HeLa cells, whereas the thiazolidine‐linked conjugate was inactive towards both cells at any time point. A HPLC‐study of the cleavage processes revealed uncontrolled degradation of the thiazolidine linked conjugate **1 a** over time, which was probably caused by reactive oxygen species produced by gossypol. On the other hand, the imine‐linked conjugate **1 b**, cleanly released gossypol over the course of multiple hours, which is an important prerequisite for targeted tumor delivery.

**Figure 1:**
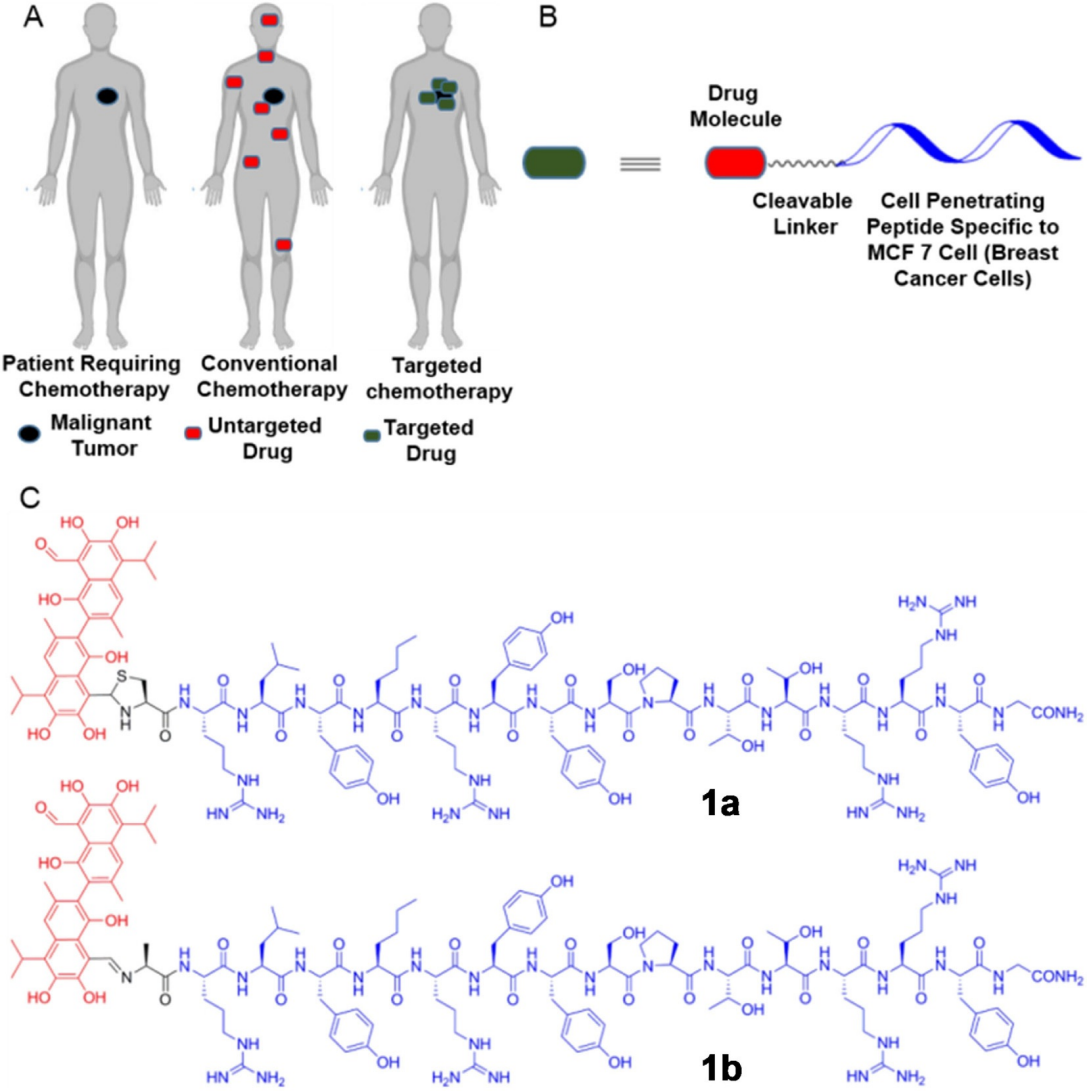
(A) The mechanism and advantage of tumor targeted drug delivery in chemotherapy. (B) Model of the designed tumor‐homing peptide–drug conjugate. (C) The chemical structures of the tumor homing peptide‐drug conjugates connected through thiazolidine and imine linkage.

Initially, a suitable linker between gossypol and the tumor‐homing cell penetrating peptide RLYMRYYSPTTRRYG had to be selected, which allows for efficient intracellular release of the anticancer drug. Although several cleavable linkers were reported for different applications, many of them are difficult to incorporate into peptide backbones, while others require specific cleavage conditions such as the involvement of enzymes, nucleophilic/basic or electrophilic/acidic reagents, reducing or oxidizing agents, photoirradiation, organometallic and metal reagents.^21^ Furthermore, these methods sometimes leave a residual moiety on the released cargo.^21^ Anticancer drugs such as doxorubicin or paclitaxel were linked through ester and amide bonds to peptides, but slow cleavage of these bonds in the cellular environment limits the activity of the drug.^22^ Disulfide linkages, which can be cleaved by glutathione in cells, were also reported. However, this strategy is not traceless and the release of the active drug can be inhibited due to drug dimer formation through disulfide bonds.^23^ The aldehyde functionality of gossypol opens up the possibility to insert a thiazolidine linkage (**1 a**). In an earlier report a thiazolidine was employed for traceless release of a drug from an antibody‐drug conjugate.^24^ An imine‐based linkage through an Ala‐modified homing peptide was also explored (**1 b**). Gossypol is known to form comparably stable conjugates with amines as well as small peptides,^25^ as the resulting Schiff's base is stabilized by two cooperative intramolecular hydrogen bonds formed by the *ortho*‐ and *meta*‐hydroxyl groups.^26^

For this work the tumor homing peptide specific to MCF‐7 cells was synthesized by Fmoc‐based solid‐phase peptide synthesis with a cysteine residue attached at the N‐terminus (Figure 2A). The methionine residue was substituted by its analogue, norleucine, to avoid oxidation. Initially, the ligation reaction was attempted between peptide **2 a** and an excess of gossypol to avoid reaction of the second aldehyde moiety. Therefore, the peptide was dissolved in 6 m Gn**⋅**HCl buffer at pH 5, followed by its addition to five equivalents of gossypol in methanol and the solution was heated at 45 °C for two hours. Consumption of the peptide was observed by HPLC analysis with emergence of two new peaks, corresponding to diastereomers formed by the axially chiral *rac*‐gossypol and the chiral peptide (Figure 2 B). The peptide–gossypol conjugates were purified by preparative HPLC followed by evaporation of methanol and lyophilization. Mass spectrometry showed a similar *m*/*z* pattern for both peaks, the main signal, however, was 32 Da less than **1 a**, which corresponds to imine **1 b** (Figure 2 C).

**Figure 2:**
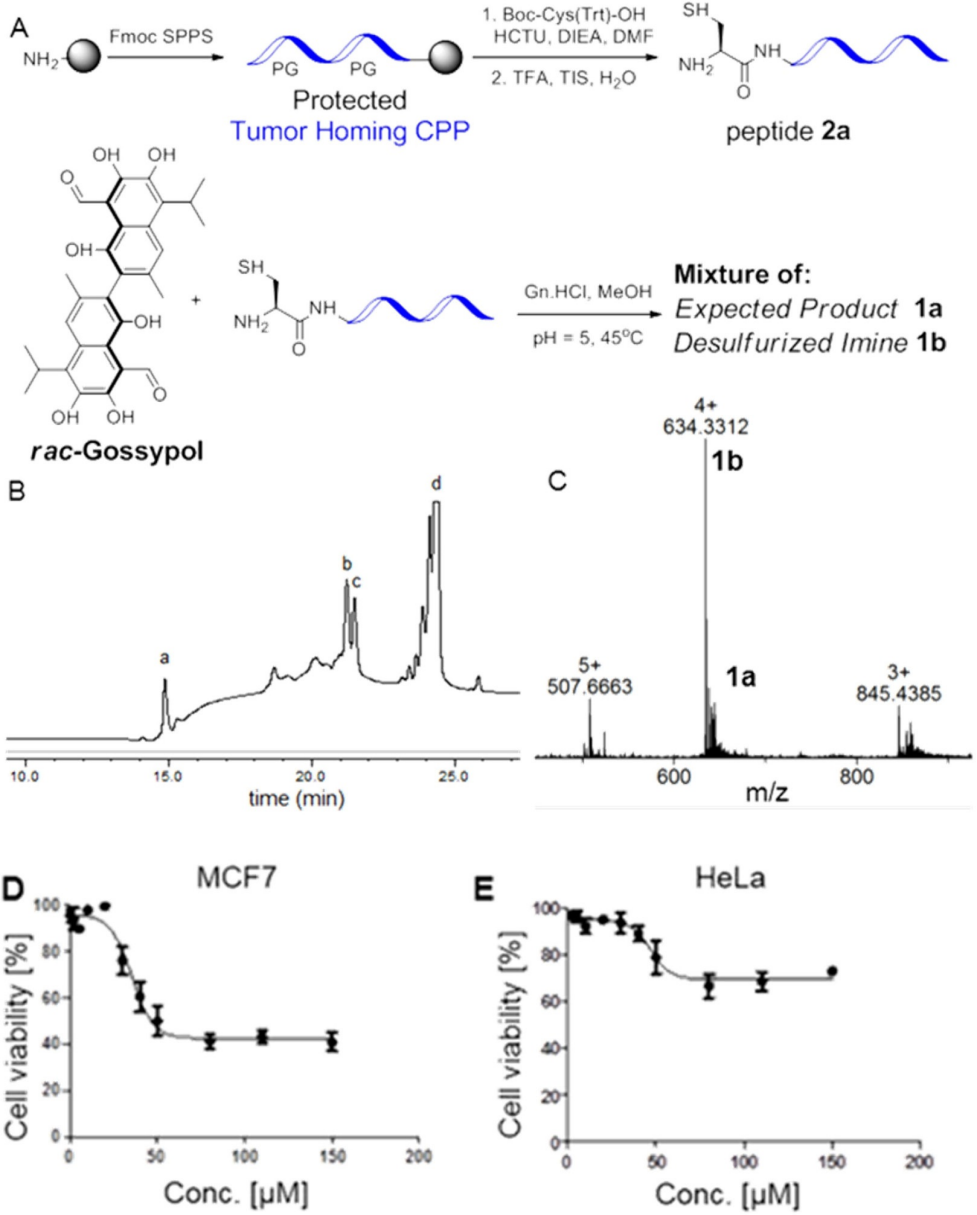
(A) Initial attempt to synthesize the peptide **2 a**–gossypol conjugate through thiazolidine ligation led to a mixture from which the active compound **1 b** was identified. (B) Analytical HPLC trace of the reaction between peptide **2 a** and gossypol at 45 °C after 2 hours with a gradient 5–100 % B in 20 min (buffer B is MeOH with 0.1 % TFA). Peak (I) corresponds to unreacted peptide; peak (II) and (III) correspond to diastereomeric ligation products, whereas peak (IV) corresponds to unreacted gossypol. (C) HRMS data of the ligated product (peak II in the HPLC spectra) after lyophilization. (D) and (E) Cell cytotoxicity assays of MCF‐7 and Hela cells treated with different concentrations of mixture **1 a/1 b** isolated from peak (II). MCF‐7 cell (D) and HeLa cell (E) treatment with the conjugation product represented by peak II. Cell viability was measured using the CellTiter 96 AQueous One Solution Cell Proliferation Assay (Promega), the absorptrion values (490 nm) corresponding to each drug concentration were obtained by subtracting the medium only control from all data points and normalizing the absorption to the highest absorption measured in each experiment. Depicted are the average values obtained from three experiments carried out in triplicate. Error bars depicture the standard deviation.

According to mass spectrometric analysis, the product obtained mainly contained **1 b** but also showed a mass corresponding to **1 a**. The cytotoxicity of both fractions was tested on MCF‐7 cells as target cells and HeLa cells as a negative control. Gossypol itself was equally potent in killing both cell lines (Figure S13, Supporting Information), while the peptide **2 a** alone was nontoxic for both (Figure S13). Gratifyingly, a specific toxicity towards MCF‐7 cells was observed for the CPP‐Gossypol conjugate mixture. Cell viability for HeLa cells for mixture (Figure 2E) was higher than for the MCF‐7 cells (Figure 2D).

Having evidence for the cell specific toxicity of peptide‐gossypol conjugates of type **1**, we turned our attention towards deducing the structure of the active conjugate. To exclude that some of the products arose from the simultaneous condensation of the second aldehyde functionality of gossypol with a nearby Arg residue (Figure S9, Supporting Information), we probed the gossypol conjugation reaction with a model tripeptide (CRL) derived from the N‐terminus of the peptide **2 a**. However, only the expected thiazolidine linked conjugate was formed (Figure S10, Supporting Information) as confirmed by mass spectrometry analyses. We also performed a ligation experiment between gossypol and Fmoc‐Arg‐OH under similar conditions to exclude condensation of the guanidine moiety with the gossypol aldehyde. No conjugation product was observed as evident from analytical HPLC (Figure S11, Supporting Information).

The molecular mass loss of 32 Da compared to the parent Cys containing peptide **1 a** (Figure S12, Supporting Information) was attributed to desulfurization of the Cys residue to Ala. As reliable thiazolidine formation under these conditions had been described before for other aldehydes,^24^ desulfurization during the ligation reaction was most likely promoted by gossypol, which is known to produce reactive oxygen species (ROS).^27^

To confirm the formation of the imine linked conjugate **1 b** through desulfurization, we synthesized **1 b** through simple ligation of gossypol and the tumor homing peptide derivative **2 b**, which was modified with an alanine residue at the N‐terminus instead of cysteine. The reaction was also conducted in MeOH and aqueous Gn**⋅**HCl‐Buffer. It afforded the peptide–drug conjugate **1 b** with a stable imine linkage as confirmed by HPLC and mass spectrometry (Figure 3, Figure S4). As in this study preparation of CPP‐gossypol conjugates of type **1** was conducted by SPPS, the available material was limited to sub mg amounts. Therefore, imine formation was confirmed by reaction of a model tripeptide NH_2_‐Ala‐Arg‐Leu‐CONH_2_ (**ARL**) and gossypol. The formation was monitored by HPLC/mass analyses (Figure S5, Supporting Information) and time‐dependent ^1^H NMR experiments (MeOD/deuterated Gn⋅HCl buffer). The proton signal at *δ*=11.05 ppm, which corresponds to the aldehyde moiety, gradually disappeared and a new signal at *δ*=9.91 ppm, corresponding to imine, appeared after three hours (Figure S7, Supporting Information). The resulting imine was isolated and fully characterized. Furthermore its structure was confirmed by HSQC (^1^H‐^13^C heteronuclear single quantum coherence spectroscopy, Figure S7, Supporting Information). As mentioned above, the unusual stability of such Schiff's bases resulting from nucleophilic addition of Gossypol with amines has been described before and can be attributed to stabilization through intramolecular hydrogen bonds as indicated in Figure 3.^25^, ^26^ The question remained, whether it was the thiazolidine conjugate **1 a** or its imine congener **1 b**, which was responsible for the encouraging selective cell toxicity observed in the initial experiments. To prevent desulfurization of the Cys residue during ligation of gossypol to peptide **2 a**, the reaction was repeated under an argon atmosphere at lower temperature (37 °C), which yielded a clean sample of **1 a** after purification by HPLC (Figure 3 c).

**Figure 3:**
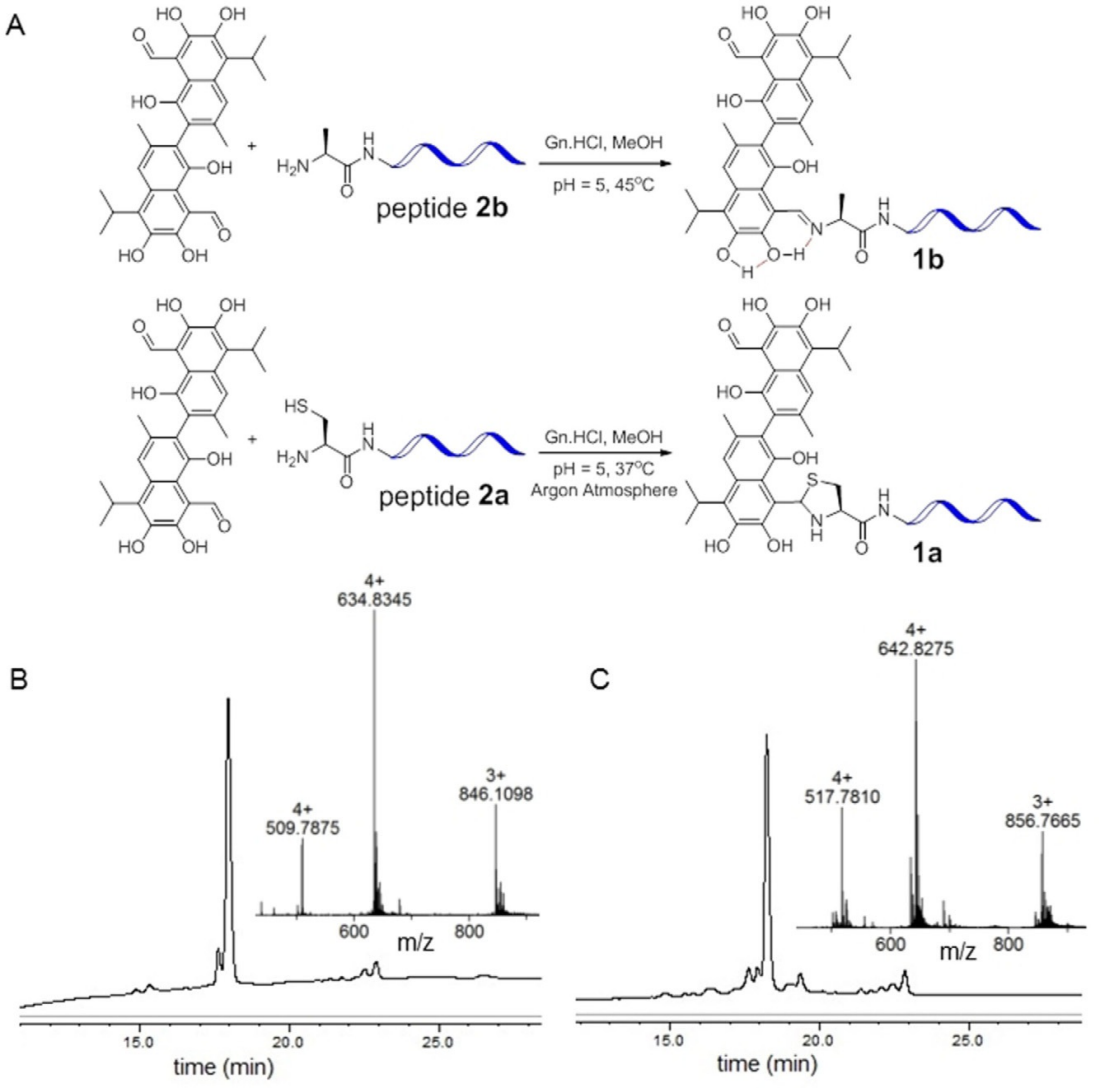
(A) Synthesis of the imine linked and thiazolidine linked conjugates. (B) Analytical HPL and mass data of the imine linked conjugate. (C) Analytical HPLC and mass data of the thiazolidine linked conjugate. (Solvent gradient for analytical HPLC was 40–100 % B in 20 min, buffer B was MeOH with 0.1 % TFA).

The cytotoxic effects of the two differently linked peptide–drug conjugates **1 a** and **1 b** were investigated in a time‐dependent cytotoxicity assay. Cell viabilities were studied after 12, 24 and 48 hours (Figure S15, Supporting Information). As shown in Figure 4, the imine linked conjugate **1 b** reduced the cell viability of MCF‐7 cells to 26 %^28^ after 48 h, whereas both HeLa cells (derived from cervical cancer) and Wi‐38 cells (normal human fibroblasts) had a much higher viability of 67 and 74 %, respectively. This underlines the cancer type selectivity of this compound. In contrast the thiazolidine linked conjugate **1 a** was inactive to both MCF‐7 and HeLa cells at any time point. Moreover, the other diastereomeric conjugates separated by HPLC for both linkages showed a similar trend of cytotoxicity, that is, the imine linked conjugate of type **1 b** was potent to selectively kill the MCF‐7 cells, while the thiazolidine linked conjugate of type **1 a** was not (Figure S14, Supporting Information).

**Figure 4:**
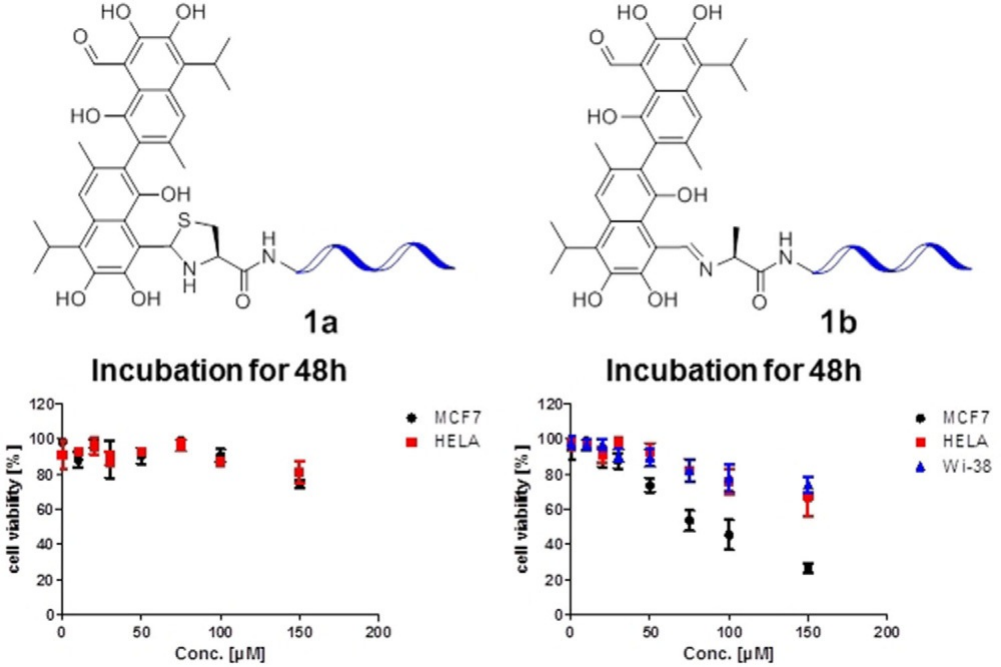
The in vitro cytotoxicity of **1 a** and **1 b** was evaluated by cell viability determination. MCF‐7, HeLa and Wi‐38 cells were treated for 48 hours with different concentrations of the two compounds. Cell viability was measured using the Cell Titer 96 AQueous One Solution Cell Proliferation Assay (Promega), the absorption values (490 nm) corresponding to each drug concentration were obtained by subtracting the medium only control from all data points and normalizing the absorption to the highest absorption measured in each experiment.

The time‐dependent gossypol release of both, the active imine based conjugate **1 b** and the inactive thiazolidine based conjugate **1 a** were studied in 6 m Gn⋅HCl buffer at pH 7 and 37 °C through time dependent HPLC analysis. The imine linked conjugate **1 b** had a half‐life of approximately 10 hours and cleanly released gossypol as well as the homing peptide **2 b** (Figure 5). On the other hand, the thiazolidine linked conjugate **1 a** decomposed to several unidentified species after two hours (Figure S17 A, Supporting Information). This observation is consistent with the observed inactivity of the thiazolidine linked conjugate in both cell lines. Assuming a similar stability for **1 b** in the cytoplasm, the measured half‐life of 10 h offers a sufficient period for intracellular accumulation of the peptide–drug conjugate **1 b** before the controlled release of the anticancer drug within MCF‐7 cells. This leads to substantial cell death after 24 and 48 h.

**Figure 5:**
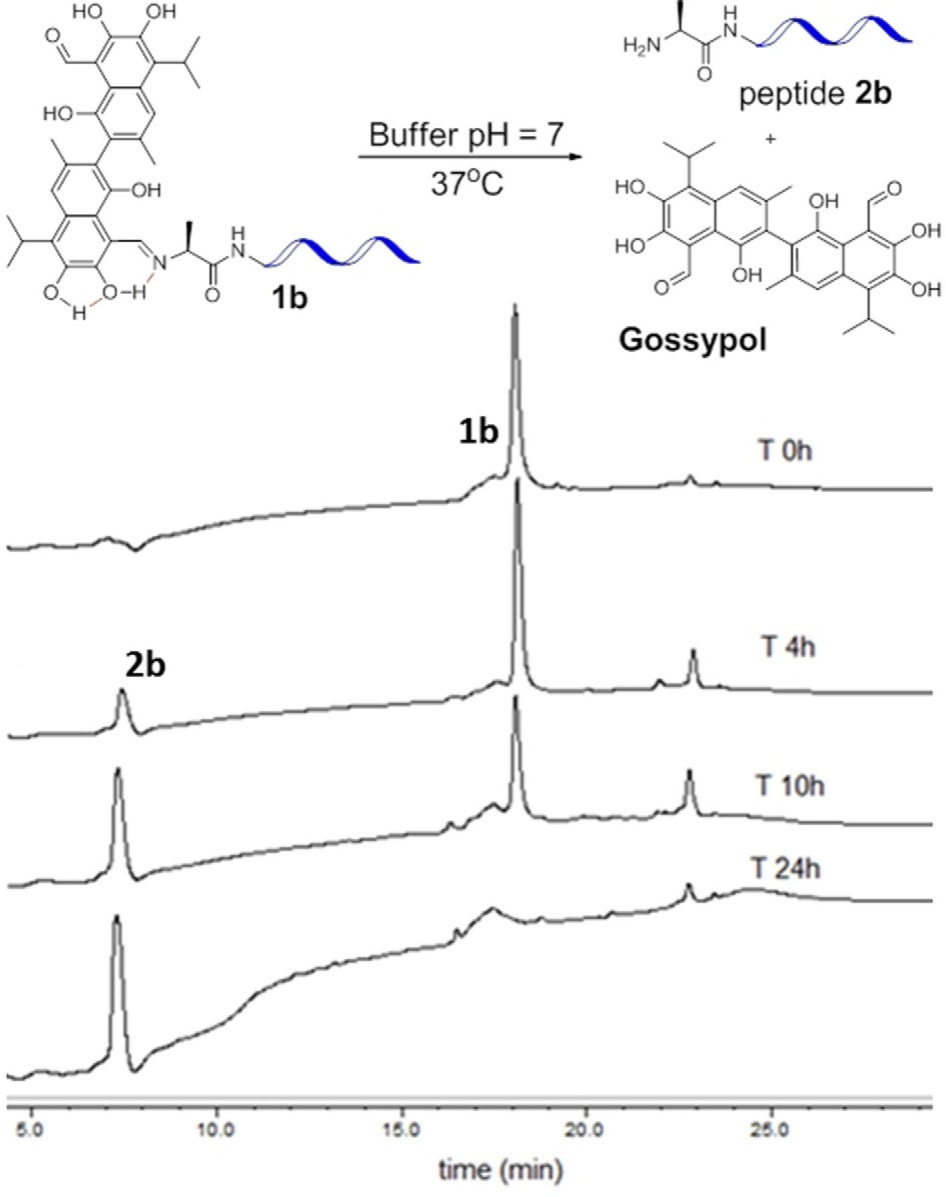
Cleavage of **1 b** in aqueous buffer at pH 7 over time. The concentration was monitored by HPLC at 2, 4, 6, 8, 10, 12 and 24 hours (Figure S17). Solvent gradient for HPLC was 40–100 % B in 20 min; buffer B was MeOH with 0.1 % TFA and UV was measured at 220 nm. Integration of the peaks corresponding to the cleaved peptide and the intact peptide‐drug conjugate revealed that 52 % of the conjugate was cleaved after 10 hours.

In summary, we have developed a cancer cell specific delivery system for gossypol by using simple Schiff's base ligation chemistry to generate a semi‐labile conjugate of Gossypol and a cancer type specific cell penetrating peptide as a vector. Imine formation between gossypol with the Alanine functionalized CPP **2 b** resulted in a potent conjugate, which killed specifically MCF‐7 breast‐cancer cells. Furthermore, the solubility of gossypol was improved, which made handling of the conjugates for cellular studies very convenient, as potentially harmful solubilizing agents like DMSO were not necessary. Importantly, the presented drug delivery strategy does not rely on any external stimulus to initiate drug release and activation. A particular advantage of this imine linkage is the convenient half‐life of 10 hours in aqueous media. Hopefully these results accelerate the applicability of gossypol particularly in tumor targeted chemotherapy. In future research cell line‐derived xenograft (CDX) mouse models should be established to evaluate the in vivo efficacy of the CPP‐gossypol conjugate **1 b**. In a broader sense, the reported approach demonstrates that cell‐penetrating peptides with tumor homing properties can be easily ligated to gossypol without the need for an additional linker. Therefore, it should be easy to expand the scope of this approach to other cancer types, for which appropriate homing peptides can be identified.

## Conflict of interest

The authors declare no conflict of interest.

## Supporting information

As a service to our authors and readers, this journal provides supporting information supplied by the authors. Such materials are peer reviewed and may be re‐organized for online delivery, but are not copy‐edited or typeset. Technical support issues arising from supporting information (other than missing files) should be addressed to the authors.

Supplementary
