# Supplementary material for: Cancer‐Cell‐Specific Drug Delivery by a Tumor‐Homing CPP‐Gossypol Conjugate Employing a Tracelessly Cleavable Linker
Source: Chemistry. 2020 Jan 30;26(14):3010–5. doi: 10.1002/chem.201905159 (PMC7079238; doi:10.1002/chem.201905159)
Supplement: Supplementary file 1 — Supplementary [file CHEM-26-3010-s001.pdf]

CHEMISTRY
A European Journal

Supporting Information

Cancer-Cell-Specific Drug Delivery by a Tumor-Homing CPP-Gossypol Conjugate Employing a Tracelessly Cleavable Linker

Suman Kumar Maity,[a] Paul Stahl,[b] Astrid Hensel,[b] Shirley Knauer,[b]
Christoph Hirschhäuser,*[a] and Carsten Schmuck†[a]

chem_201905159_sm_miscellaneous_information.pdf

Cancer-Cell Specific Drug Delivery by a Tumor Homing CPP-Gossypol Conjugate employing a Tracelessly Cleavable Linker

Suman Kumar Maity, Paul Stahl, Astrid Hensel, Shirley Knauer, Christoph Hirschhäuser* and Carsten Schmuck†

SUPPORTING INFORMATION

Table of Contents

Experimental Section S2

Materials and methods S2

List of the protected amino acids used in peptides synthesis: S2

Synthesis of peptide 2a S3

Synthesis of thiazolidine linked peptide-gossypol conjugates 1a S5

Synthesis of peptide 2b S8

Synthesis of imine linked peptide-gossypol conjugates 1b S9

Ligation of model tripeptide Ala-RL and gossypol S12

Initial Attempt at Ligation of Peptide 2a and Gossypol Leading to an Active Product Mixture S17

Control Experiments for Structure Elucidation S19

Ligation of Cys-RL with gossypol S19

Ligation of Fmoc-Arg-OH and gossypol S21

Cell culture and cytotoxicity assay S23

Kinetics study of the cleavage of the imine and thiazolidine linkages S27

Experimental Section

Materials and methods

SPPS was carried out manually in a glass reaction vessel, equipped with teflon filters on Rink amide resin. Analytical grade *N,N*dimethylformamide (DMF) was purchased from Fisher Chemical. Rink amide resin was purchased from Fluorochem Ltd, protected amino acids were purchased from GL Biochem and activating reagents [(6-chlorobenzotriazol-1-yl)oxy-(dimethylamino)methylidene]-dimethylazanium hexafluorophosphate (HCTU) and 1-[Bis(dimethylamino)methylene]-1H-1,2,3- triazolo[4,5-b]pyridinium 3-oxid hexafluorophosphate (HATU)] were purchased from Iris Biotech. Chemicals were purchased from Aldrich, and Alfa Aesar. Analytical HPLC was performed on a Thermo instrument (Dionex Ultimate 3000) Dionex containing the following components: P680 HPLC pump, ASI-100 Automated Sample Injector and UVD 340U detector. A YMC ODS-AQ C18 column (15 cm × 3.0 mml.D., S-5 μm, 12 nm) was utilized at a flow rate of 0.43 mL/min for analytical HPLC. Ultrapure water and HPLC-grade solvents were used as eluents. Detection was achieved with the help of a UV detector. Preparative HPLC was performed on a Knauer instrument using YMC-Actus ODSA C18 (150×20 mml.D. S-5 μm, 12 nm) column, at flow rate of 15 mL/min. ^{1}H- and ^{13}C-NMR spectra were recorded on DRX 300 and 500 MHz spectrometers from Bruker at ambient temperature. All synthetic products were purified by HPLC and characterized by mass spectrometry using a Bruker BioTOF III. Mass analysis was performed with Bruker Data Analysis 4.1 program. Lyophilization was carried out with an Alpha 1-4 2D plus freeze drying apparatus from Christ. Determination of pH values was carried out with a pH meter 766 Calimatic from Knick.

Buffer A: 0.1% TFA in water; buffer B: 0.1% TFA in methanol.

List of the protected amino acids used in peptides synthesis:

Fmoc-Gly-OH, Fmoc-Ala-OH, Fmoc-Leu-OH, Fmoc-Nle-OH, Fmoc-Arg(Pbf)-OH, Fmoc-Tyr(^{t}Bu)-OH, Fmoc-Thr(^{t}Bu)-OH, Boc-Cys(Trt)-OH, Fmoc-Pro-OH.

Synthesis of peptide 2a

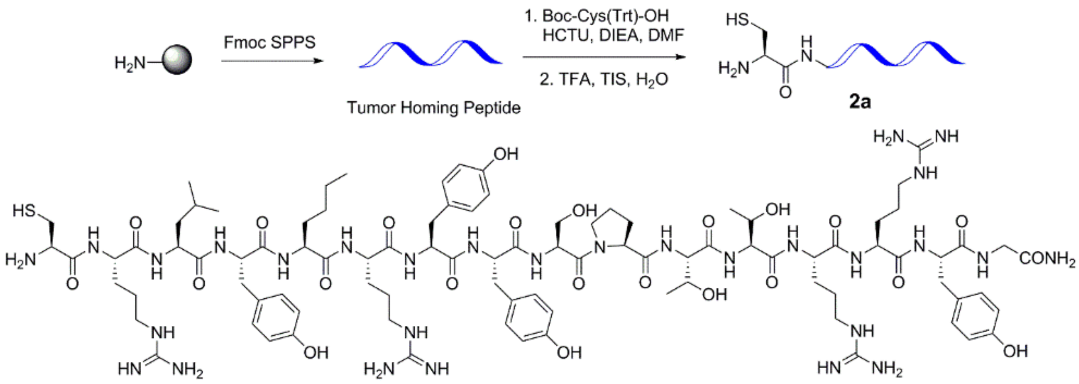

2a

The synthesis was carried out using Fmoc-SPPS on Rink amide resin (0.43 mmol/g, 0.2 mmol scale). Unless mentioned otherwise, peptide synthesis was performed in presence of 4 equiv of amino acid, HCTU and 8 equiv of *N,N'*-diisopropylethylamine (DIEA) at room temperature for 45 min. The pre-swollen resin was treated with 20% piperidine in DMF (3-5-3min) to remove the Fmoc-protecting group. The amino acids were coupled in manual peptide synthesis flask on an orbital shaker. Double coupling was performed for Fmoc-Arg(Pbf)-OH, using HCTU. Fmoc-Thr(^{t}Bu)-OH and Fmoc-Pro-OH were coupled with HATU for 1 hour. Fmoc-Nle-OH was used in place of Fmoc-Met-OH to avoid oxidation. Boc-Cys(trt)-OH was coupled at the N-terminus.

Deprotection and cleavage from the resin: The resin was washed with DMF, MeOH, DCM and dried. The peptide was cleaved using a TFA:triisopropylsilane (TIS):water (95:2.5:2.5) mixture for 2 h. The cleavage mixture was filtered and the combined filtrate was added dropwise to a 10-fold volume of cold ether and centrifuged. The precipitated crude peptide was dissolved in acetonitrile-water (1:1) and further diluted to ~30% with water and lyophilized. The HPLC analysis was carried out on a C18 analytical column using a gradient of 5-100% B over 20 min (t = 14.57 min). For preparative HPLC, 20-100% B over 60 min gradient was used to purify the peptide **2a** to the desired product in ~60% yield. **MS m/z (ESI$^+$):** 517.8 (M+4H$^+$, 100%), 690 (M+3H$^+$, 26%).

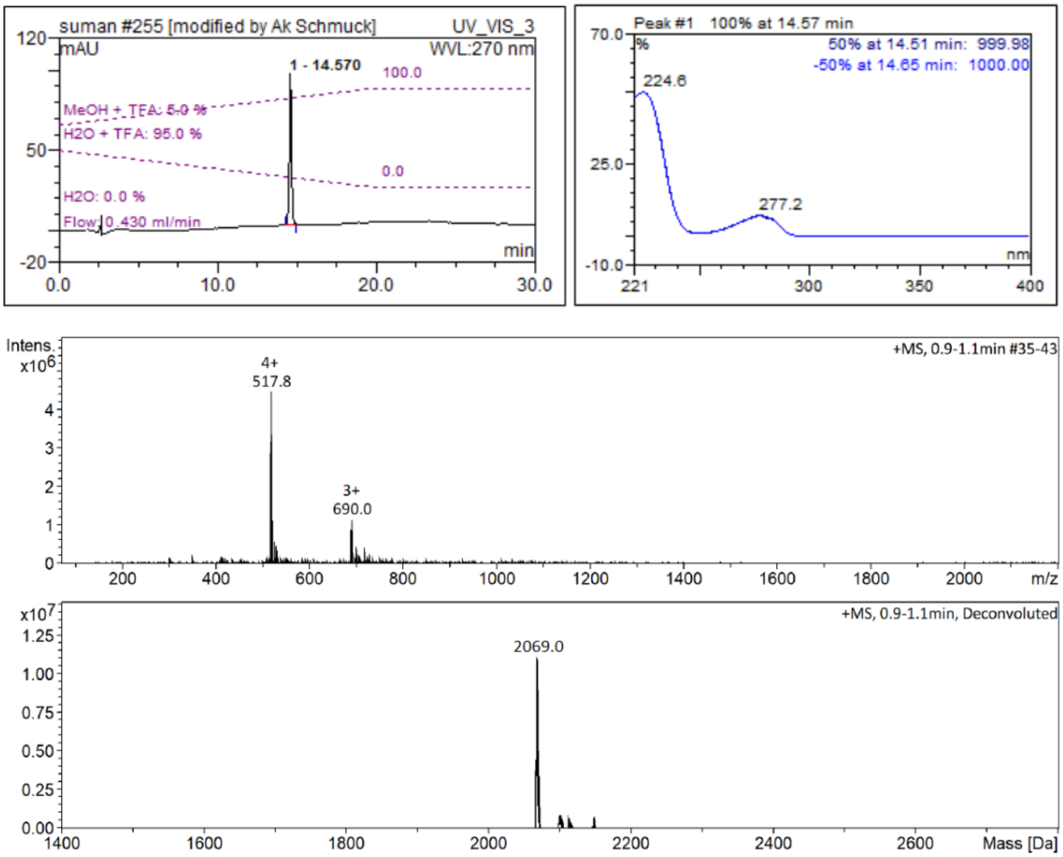

Figure S1: Analytical HPLC and mass analysis of the purified peptide **2a** with the observed mass 2069.0 Da, calcd 2070.0 Da.

Synthesis of thiazolidine linked peptide-gossypol conjugates 1a

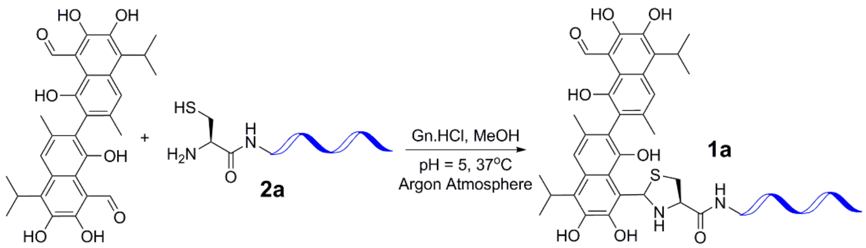

Peptide **2a** (5 mg, 2.4 × 10^{-3} mmol) was dissolved in 150 µL of argon purged 6 M Gn·HCl, 200 mM phosphate buffer at pH 5 in a reaction vial (Eppendorf). In another reaction vial, gossypol (6.25 mg, 12× 10^{-3} mmol) was dissolved in 500 µL argon purged methanol. The peptide **2a** solution was added to the gossypol solution in methanol and the reaction mixture was incubated at 37 °C for 2h under argon atmosphere. Monitoring the progress of the reaction by analytical HPLC using C18 column with a gradient of 40-100% buffer B over 30 min revealed the appearance of peak a (t = 16.65 min) and peak b (t = 18.23 min). After completion of the reaction the reaction mixture was diluted with 500 µL MeOH : H_2O (1:1) and subjected to purification by preparative HPLC with a gradient 50-100% buffer B over 60 min. After collecting peak a and peak b, methanol was evaporated by rotary evaporator and the concentrated solution was lyophilized to afford thiazolidine linked peptide-gossypol conjugates **1a** (0.41 mg, 6.6% yield for peak a; 0.62 mg 10% yield for peak b). **MS m/z (ESI$^+$):** Peak a :517.7 (M+4H$^+$, 67%, corresponds to peptide **2a**), 642.8 (M+4H$^+$, 100%), 856.7 (M+3H$^+$, 21.6%); **MS m/z (ESI$^+$):** Peak b: 517.7 (M+4H$^+$, 70%, corresponds to peptide **2a**), 642.8 (M+4H$^+$, 100%), 856.7 (M+3H$^+$, 13.5%), 1284.6 (M+2H$^+$, 2.7%)

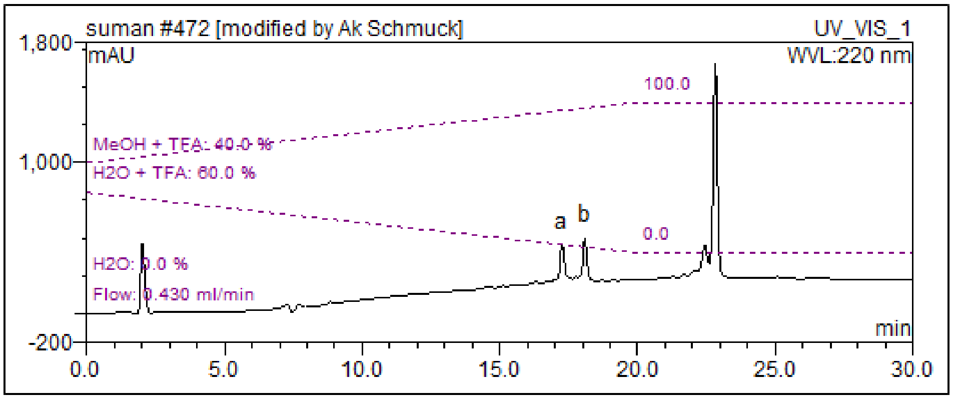

Figure S2A: Analytical HPLC of the crude product of the ligation reaction affording thiazolidines **1a**. For HPLC traces of the isolated products see Figure S2B and Figure S2C.

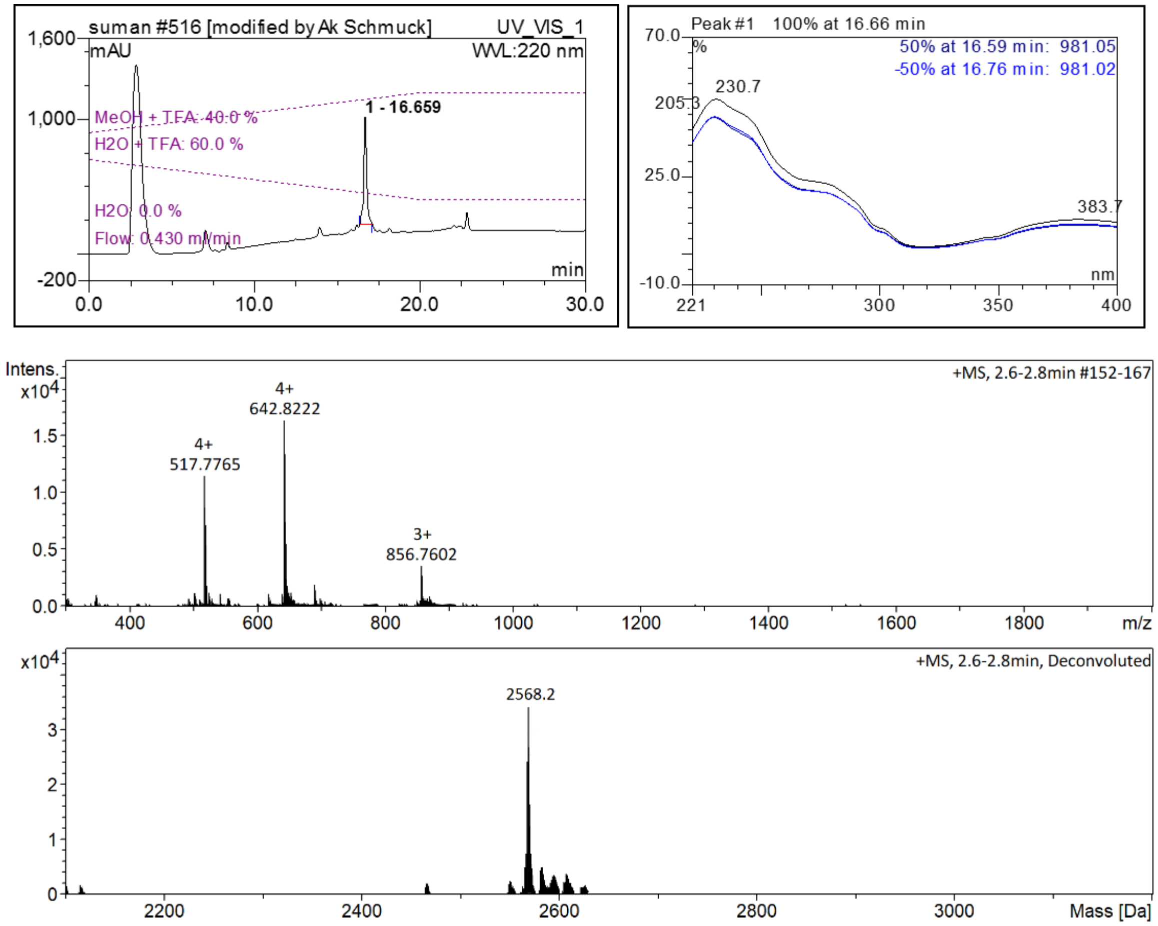

Figure S2B: Analytical HPLC and mass analysis of isolated peak **a** of thiazolidine linked conjugate with the observed mass 2568.2 Da, calcd 2569.2 Da.

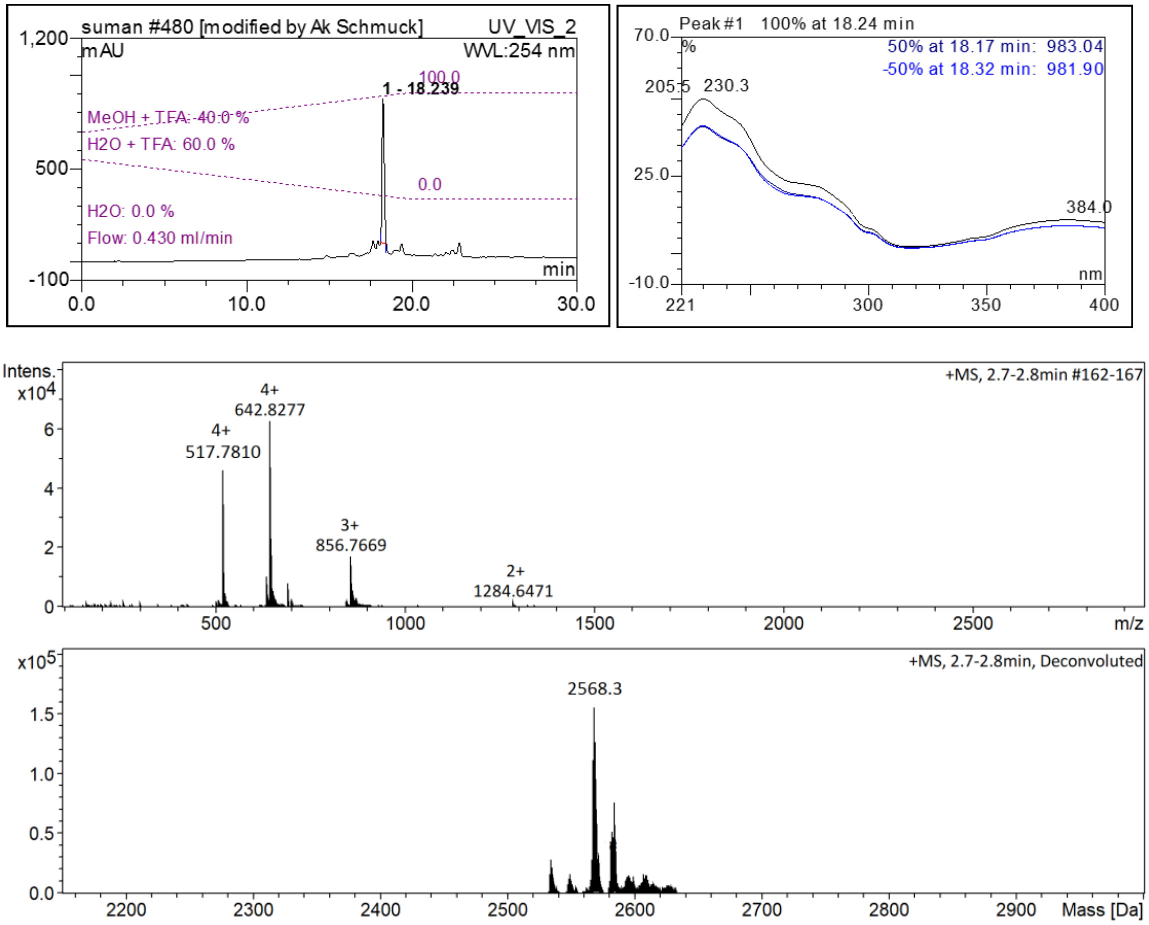

(C)

Figure S2C: Analytical HPLC and mass analysis of isolated peak **b** of thiazolidine linked conjugate 2568.3 Da, calcd 2569.2 Da.

Synthesis of peptide 2b

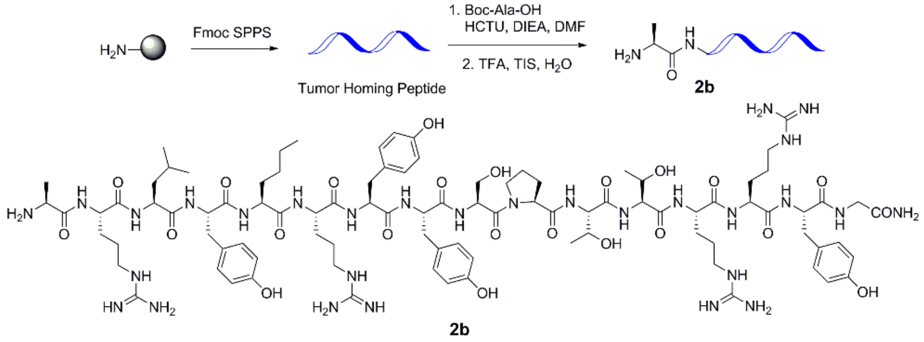

Peptide **2b** was synthesized similarly as peptide **2a** by finally coupling Boc-Ala-OH at the N-terminus instead of Boc-Cys(trt)-OH. The HPLC analysis of the purified peptide was carried out on a C18 analytical column using a gradient of 40-100% B over 20 min (t = 7.49 min). For preparative HPLC, 20-100% B over 60 min gradient was used to purify the peptide **2a** to the desired product in ~58% yield. **MS m/z (ESI$^+$):** 509.5 (M+4H$^+$, 100%), 679.7 (M+3H$^+$, 16.2%).

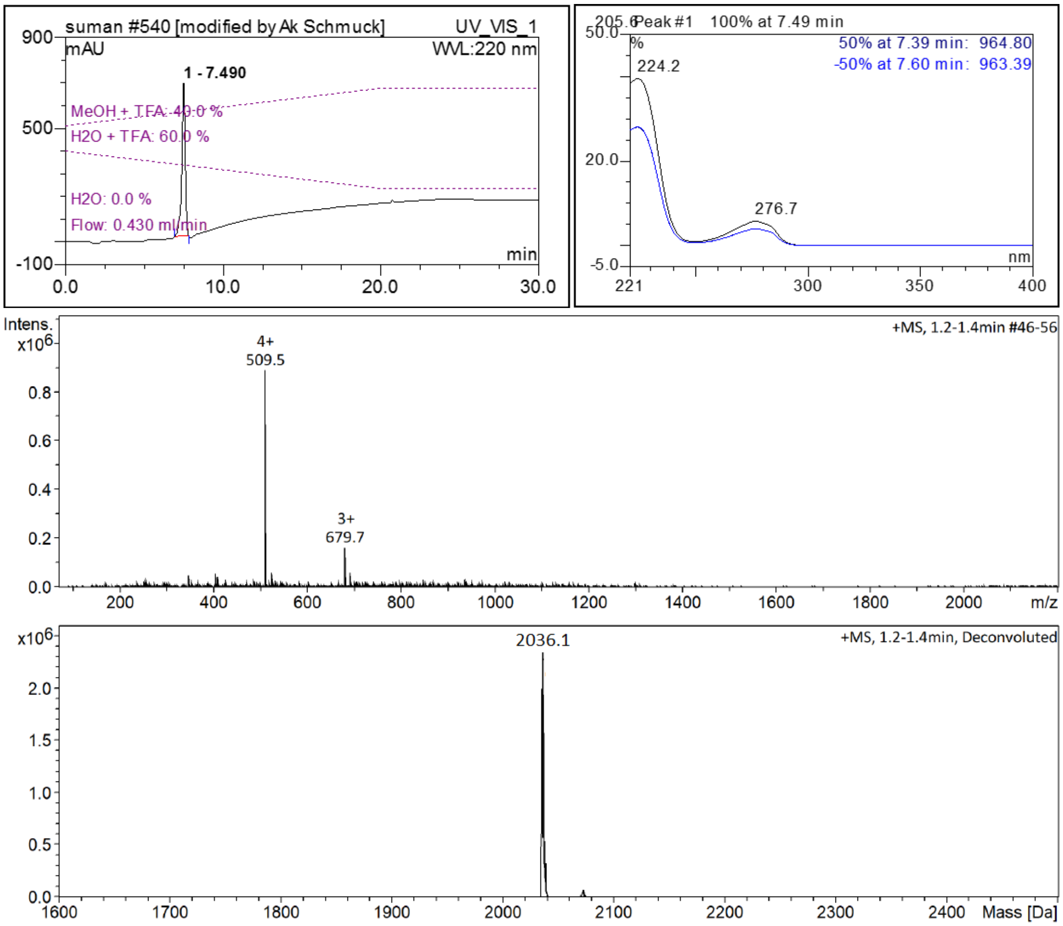

Figure S3: Analytical HPLC and mass spectra of purified peptide **2b** with observed mass 2036.1 Da, calcd 2038.1 Da.

Synthesis of imine linked peptide-gossypol conjugates 1b

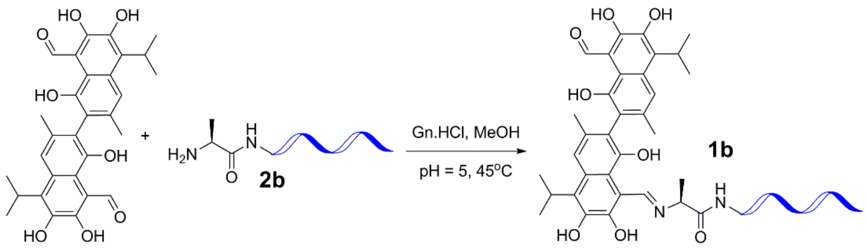

Peptide **2b** (5 mg, 2.4 × 10^{-3} mmol) was dissolved in 150 µL of 6 M Gn·HCl, 200 mM phosphate buffer at pH 5 in a reaction vial (Eppendorf). In another reaction vial, gossypol (6.25 mg, 12× 10^{-3} mmol) was dissolved in 500 µL methanol. The peptide **2b** solution was added to the gossypol solution in methanol and the reaction mixture was incubated at 45 °C for 2h. Monitoring the progress of the reaction by analytical HPLC using C18 column with a gradient of 40-100% buffer B over 30 min revealed the appearance of peak a (t = 17.29 min) and peak b (t = 17.95 min). After completion of the reaction the reaction mixture was diluted with 500 µL MeOH : H_2O (1:1) solution and subjected to purification by preparative HPLC with a gradient 50-100% buffer B over 60 min. After collecting peak a and peak b, methanol was evaporated by rotary evaporator and the concentrated solution was lyophilized to afford the imine linked peptide-gossypol conjugates **1b** (0.36 mg, 5.8% yield for peak a; 0.57 mg 9.3% yield for peak b). **MS m/z (ESI$^+$):** Peak a: 509.7 (M+4H$^+$, 50%, corresponds to peptide **2b**), 634.8 (M+4H$^+$, 100%), 846.1 (M+3H$^+$, 25%); **MS m/z (ESI$^+$):** Peak b: 509.7 (M+4H$^+$, 40%, corresponds to peptide **2b**), 634.8 (M+4H$^+$, 100%), 846.1 (M+3H$^+$, 42%). *In order to further confirm formation of **1b**, the reaction was repeated with peptide ARL and the resulting product fully characterized (pages S12-S16).*

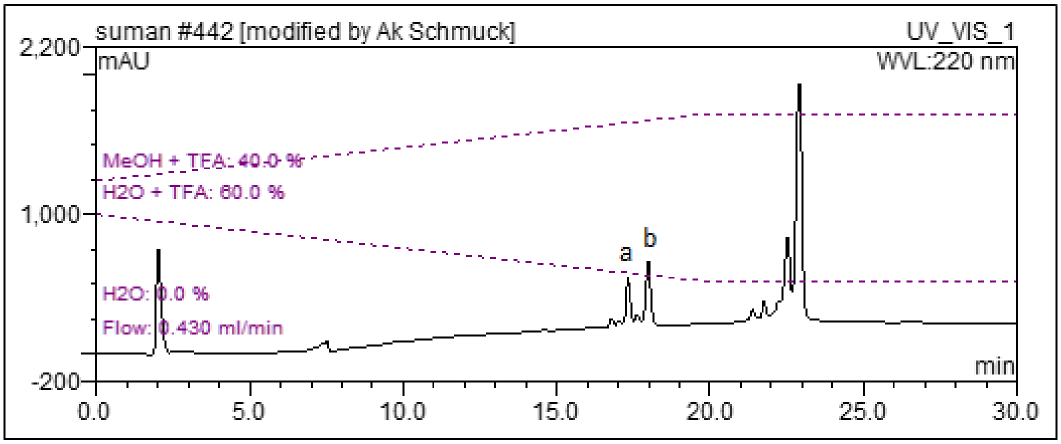

Figure S4A: Analytical HPLC of the crude product of the ligation reaction affording imine linked conjugates. For HPLC traces of the isolated products see Figure S4B and Figure S4C.

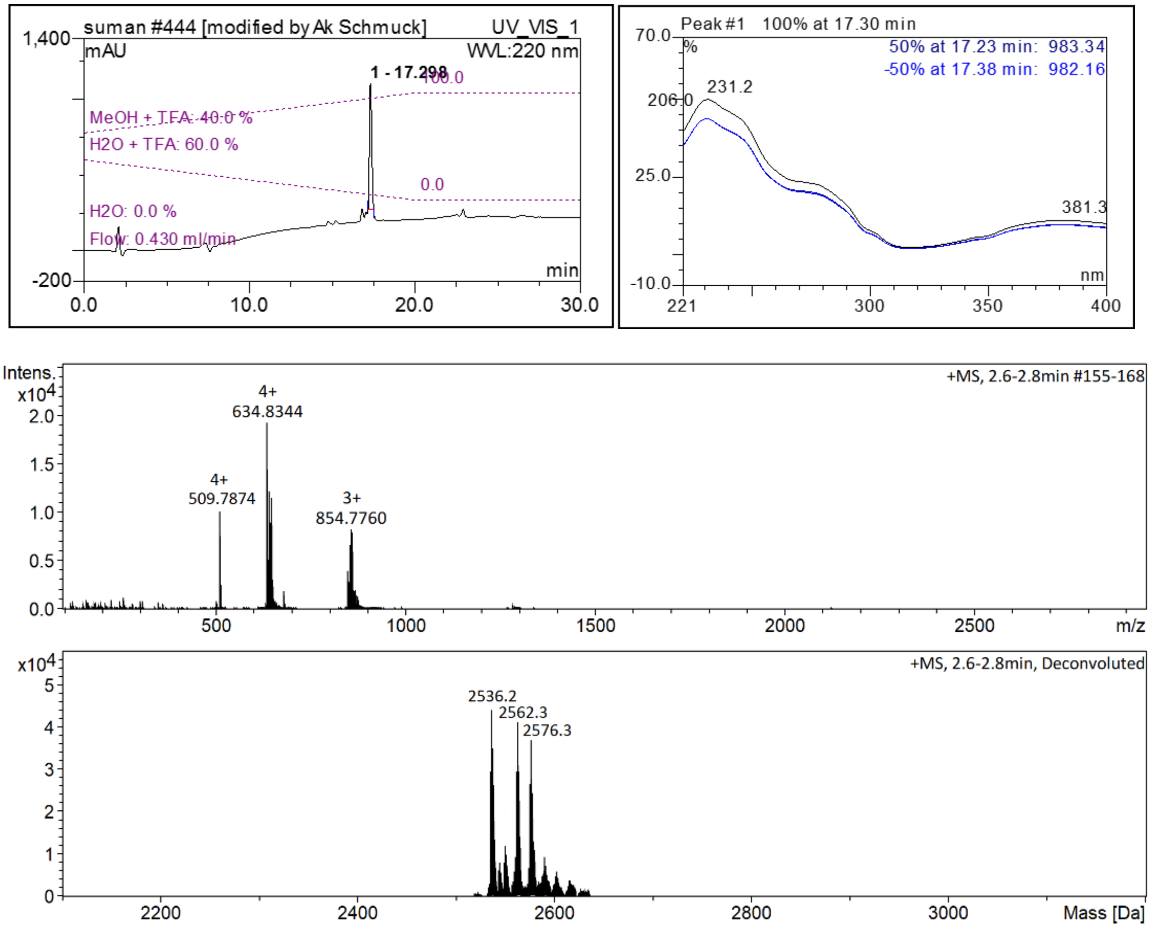

Figure S4B: Analytical HPLC and mass analysis of isolated peak a of conjugate **1b** with the observed mass 2536.2 Da, calcd 2537.2 Da. The observed mass corresponds to 2562.3 Da may be owing to the oxidation of gossypol to gossypolone moiety, calcd 2565.2 Da.

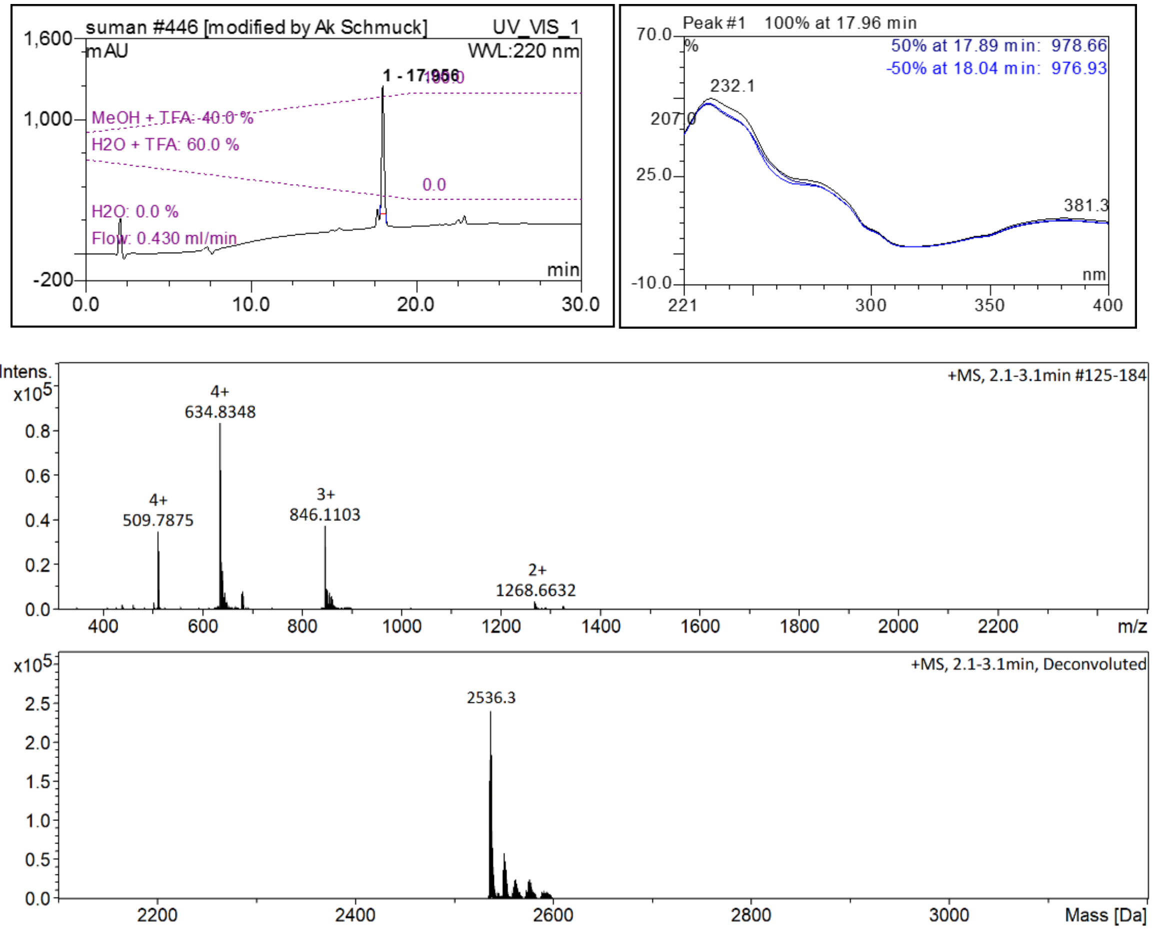

(C)

Figure S4C: Analytical HPLC and mass data of isolated peak b of conjugate **1b** with the observed mass 2536.3 Da, calcd 2537.2 Da.

Ligation of model tripeptide Ala-RL and gossypol

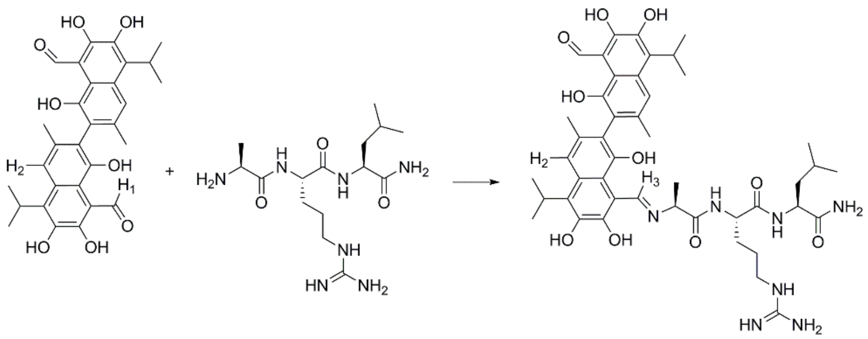

Model tripeptide Ala-RL was synthesized by Fmoc based SPPS. The HPLC analysis was carried out on a C18 analytical column using a gradient of 5-100% B over 20 min (t = 9.11 min). For preparative HPLC, 20-100% B over 60 min gradient was used to purify the peptide to the desired product in ~65% yield. **MS m/z (ESI$^+$):** 358.2 (M+1H$^+$, 100%).

Ala-RL (3 mg, 8.3×10^{-3} mmol) was dissolved in 100 μL of 6 M Gn·HCl, 200 mM phosphate buffer at pH 5 in a reaction vial (Eppendorf). In another reaction vial, gossypol (6 mg, 11.5×10^{-3} mmol) was dissolved in 500 μL methanol. The peptide solution was added to the gossypol solution in methanol and the reaction mixture was incubated at 45 ºC for 2h. Monitoring the progress of the reaction by analytical HPLC using C18 column with a gradient of 40-100% buffer B over 30 min revealed the appearance of peak a (t = 21.32 min) and peak b (t = 21.70 min). After completion of the reaction the reaction mixture was diluted with 500 μL MeOH : H_2O (1:1) solution and subjected to purification by preparative HPLC with a gradient 50-100% buffer B over 60 min. After collecting peak a and peak b, methanol was evaporated by rotary evaporator and the concentrated solution was lyophilized to afford the imine linked model peptide-gossypol conjugates (0.32 mg, 4.2% isolated yield for peak a; 0.40 mg, 5.5% isolated yield for peak b). **MS m/z (ESI$^+$):** Peak a: 858.4 (M+1H+, 100%, calc. 858.4); **MS m/z (ESI$^+$):** Peak b: 858.4 (M+1H$^+$, 100%, calc. 858.4).

The formation of imine linked conjugate was confirmed by proton and carbon NMR of the reaction mixture. **^{1}H NMR** (300 MHz, MeOD/deuterated Gn.DCl buffer, δ ppm): 11.07 (s, 1H), 9.91 (s,1H), 7.66 (s, 2H), 4.42-4.46 (m, 2H), 4.34-4.39 (m, 1H), 4.07-4.13 (m, 1H), 3.88-3.96 (m, 2H), 3.22-3.27 (t, 2H, J = 6 Hz), 2.18 (s, 3H), 2.00 (s, 3H), 1.55-1.91 (m, 7H), 1.50-1.55 (m, 15H), 0.90-0.97 (m, 6H). **^{13}C NMR** (125 MHz, MeOD/deuterated Gn.DCl buffer, δ ppm):

210.22, 177.67, 175.83, 173.67, 171.45, 160.14, 158.71, 136.14, 135.42, 130.10, 126.39, 121.17, 119.23, 117.29, 113.35, 110.23, 54.62, 53.25, 50.2942.11, 33.13, 30.03, 28.21, 26.25, 26.03, 23.63, 22.01, 21.14, 17.84. ; **FT-IR** of isolated peak b: (ATR) [cm^{-1}]: 3783.65, 3353.6, 2962.13, 2927.41, 2873.42, 1664.27, 1616.06, 1438.64, 1380.78, 1301.72, 1245.79, 1191.79, 1137.8, 1058.73, 838.883, 800.314, 771.387, 719.318.

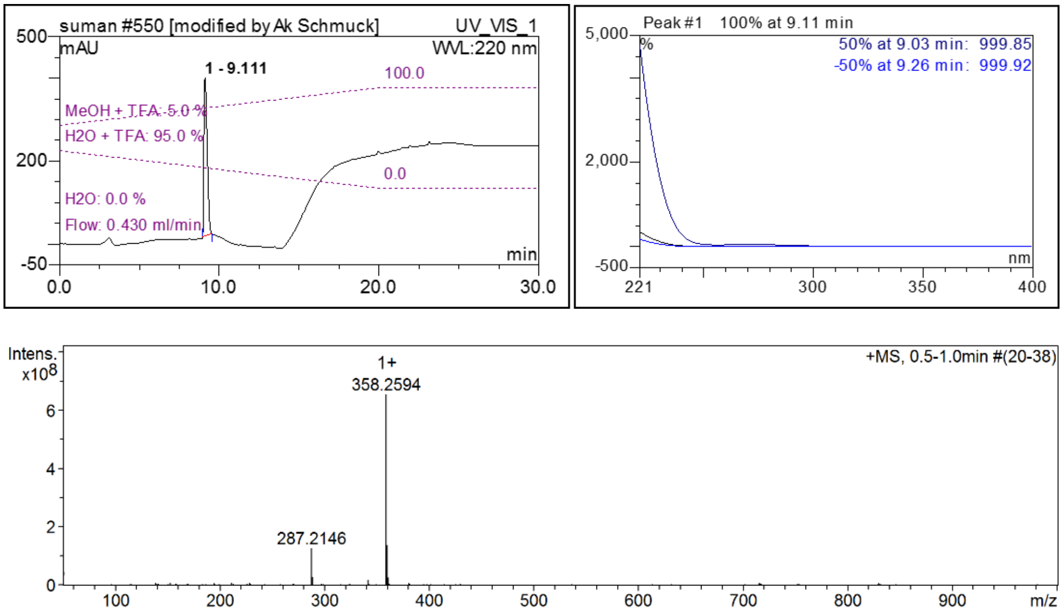

Fig S5A: Analytical HPLC and mass spectra of purified ARL tripeptide.

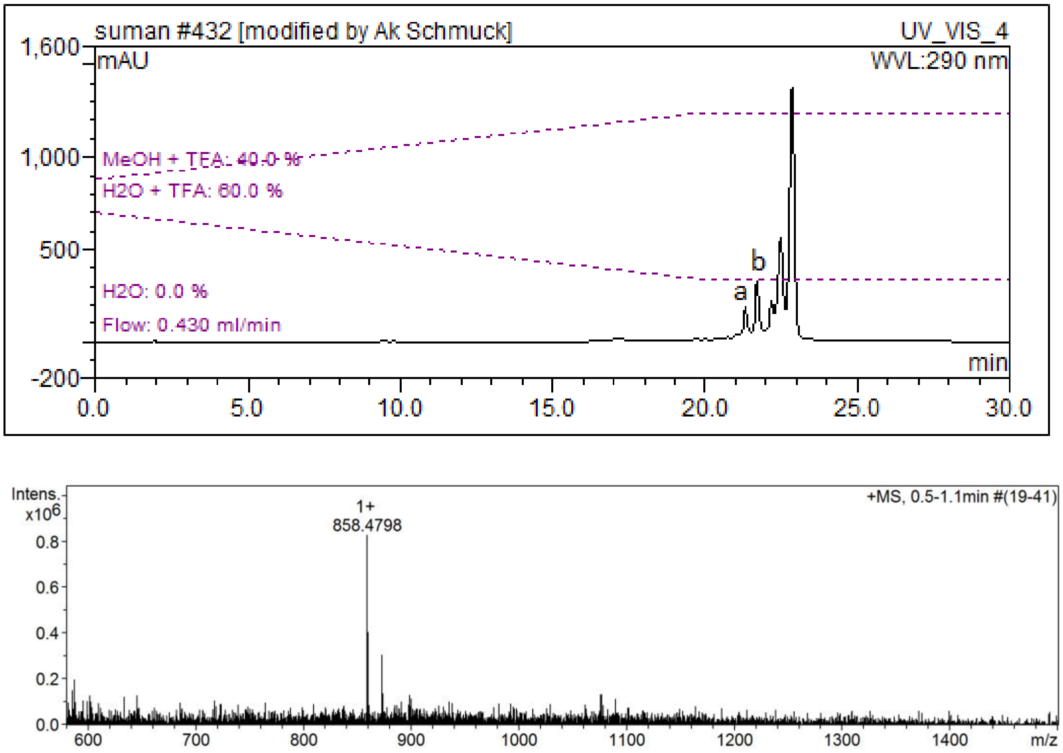

(B)

Fig S5B: Analytical HPLC and mass spectra of ligation reaction between ARL and gossypol and the mass of the isolated product b.

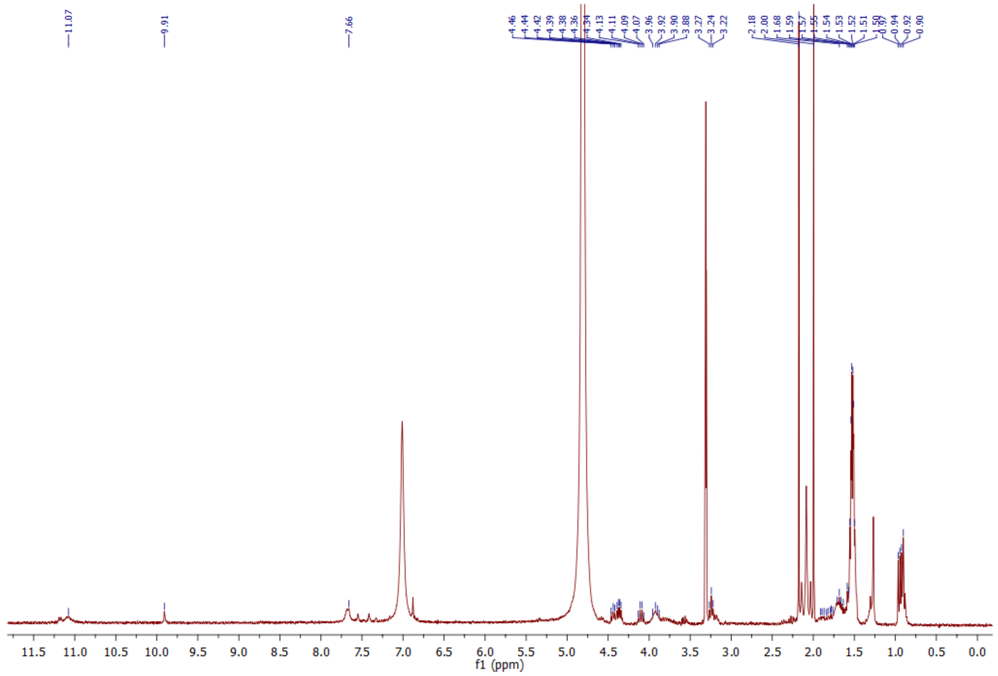

(A)

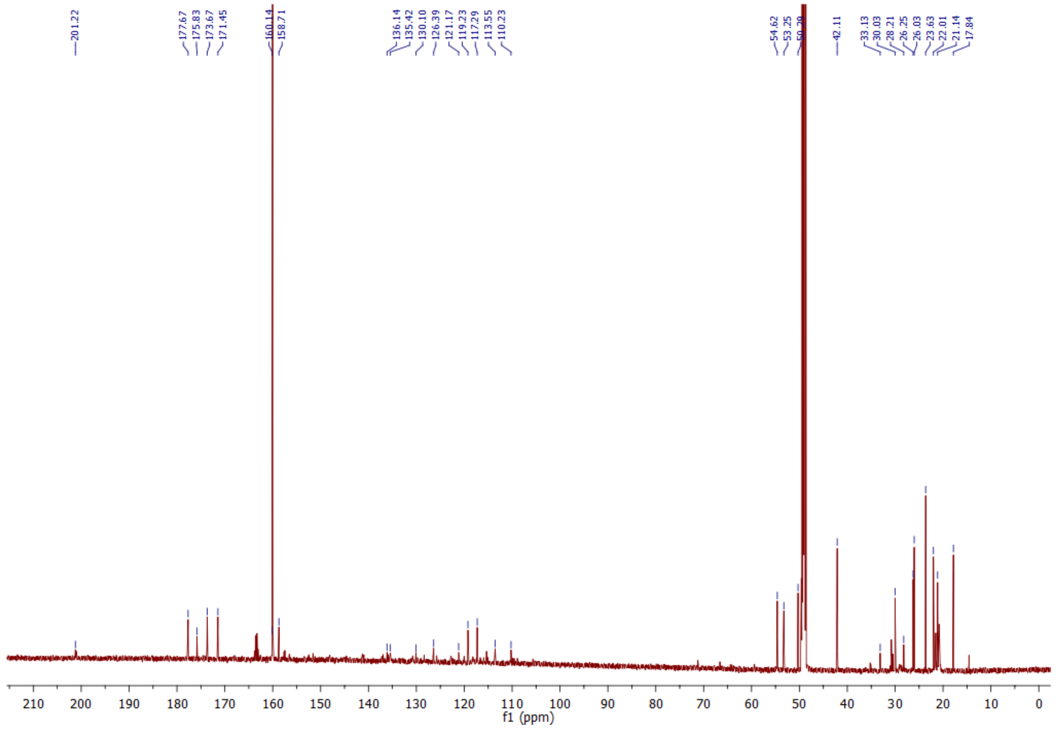

(B)

Figure S6: (A) ^{1}H NMR (300 MHz) and (B) ^{13}C (125 MHz) spectrum of the ligation reaction between model tripeptide ARL and gossypol after 3H in MeOD/deuterated Gn.DCl buffer.

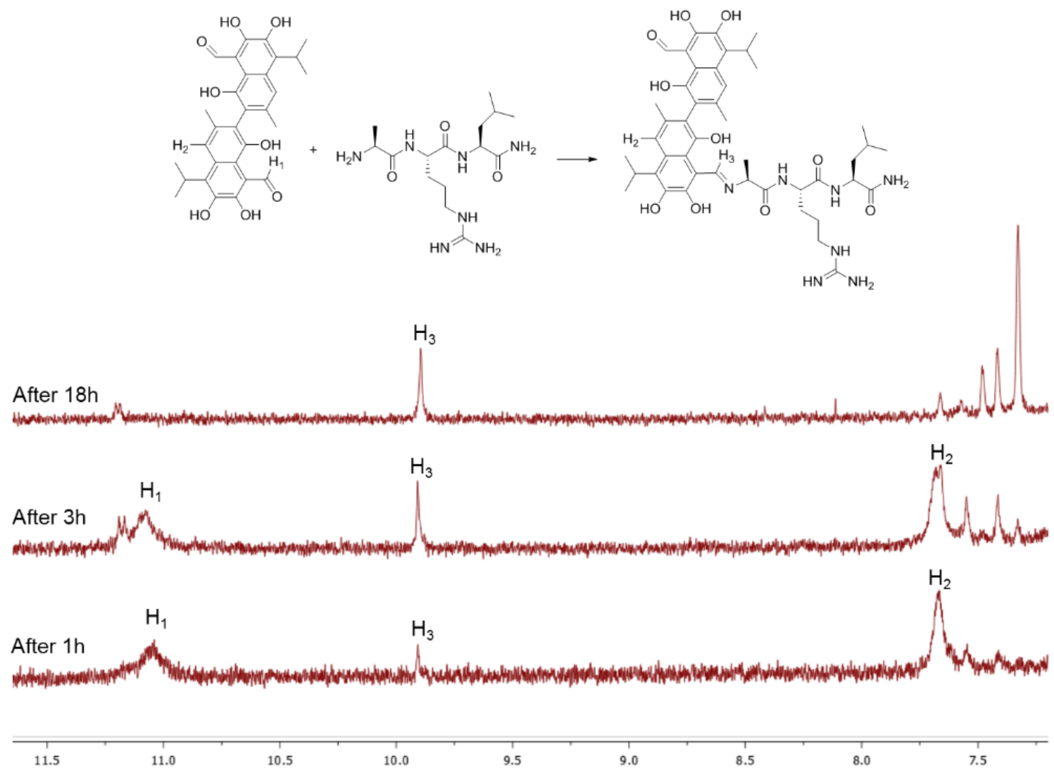

(A)

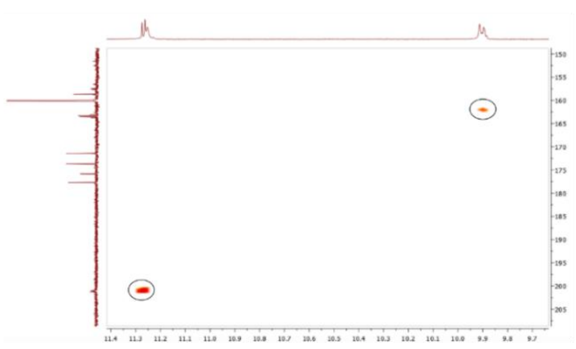

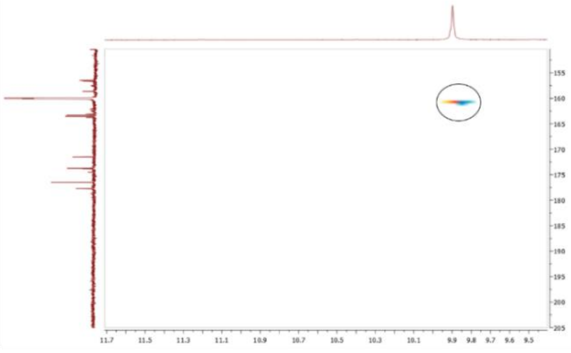

(B) (C)

Figure S7: (A) Part of time dependent ^{1}H NMR (300 MHz) spectra of ligation reaction between model tripeptide ARL and gossypol in MeOD/deuterated Gn.DCl buffer. (B) ^{1}H-^{13}C heteronuclear single quantum coherence spectrum (HSQC) after 1h, and (C) ^{1}H-^{13}C HSQC spectrum after 18h. The HSQC signal corresponding to aldehyde disappeared with the emergence of imine signal.

Initial Attempt at Ligation of Peptide 2a and Gossypol Leading to an Active Product Mixture

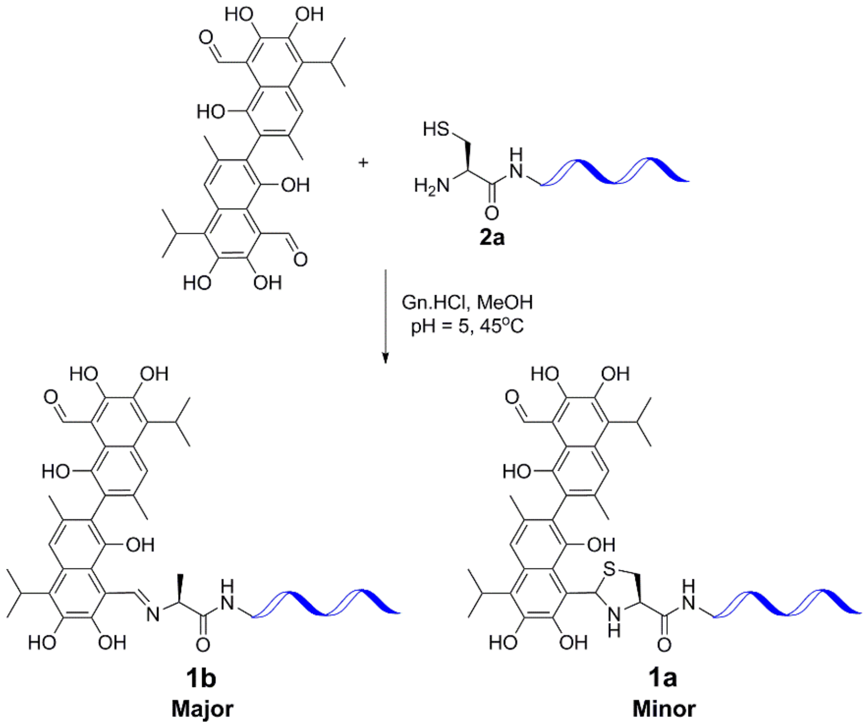

Peptide **2a** (5 mg, 2.4 × 10^{-3} mmol) was dissolved in 150 µL of 6 M Gn·HCl, 200 mM phosphate buffer at pH 5 in a reaction vial (Eppendorf). In another reaction vial, gossypol (6.25 mg, 12× 10^{-3} mmol) was dissolved in 500 µL methanol. The peptide **2a** solution was added to the gossypol solution in methanol and the reaction mixture was incubated at 45 ºC for 2h. Monitoring the progress of the reaction by analytical HPLC using C18 column with a gradient of 5-100% buffer B over 30 min revealed the appearance of peak b (t = 21.22 min) and peak c (t = 21.49 min) (c.f. Scheme 1). After completion of the reaction the reaction mixture was diluted with 500 µL MeOH : H_2O (1:1) solution and subjected to purification by preparative HPLC with a gradient 50-100% buffer B over 60 min. After collecting peak b and peak c, methanol was evaporated by rotary evaporator and the concentrated solution was lyophilized to afford the mixtures of peptide-gossypol conjugates **1a** and **1b** (0.53 mg, 8.3% yield for peak a; 0.35 mg 5.4% yield for peak b). **MS m/z (ESI$^+$):** Peak a: 634.3 (M+4H$^+$, 100%), 642.8 (M+4H$^+$, 23%), 845.4 (M+3H$^+$, 23%), 858.7 (M+3H$^+$, 10%); **MS m/z (ESI$^+$):** Peak b: 634.3 (M+4H$^+$, 100%), 642.8 (M+4H$^+$, 32%), 845.4 (M+3H$^+$, 11%), 858.7 (M+3H$^+$, 3.7%)

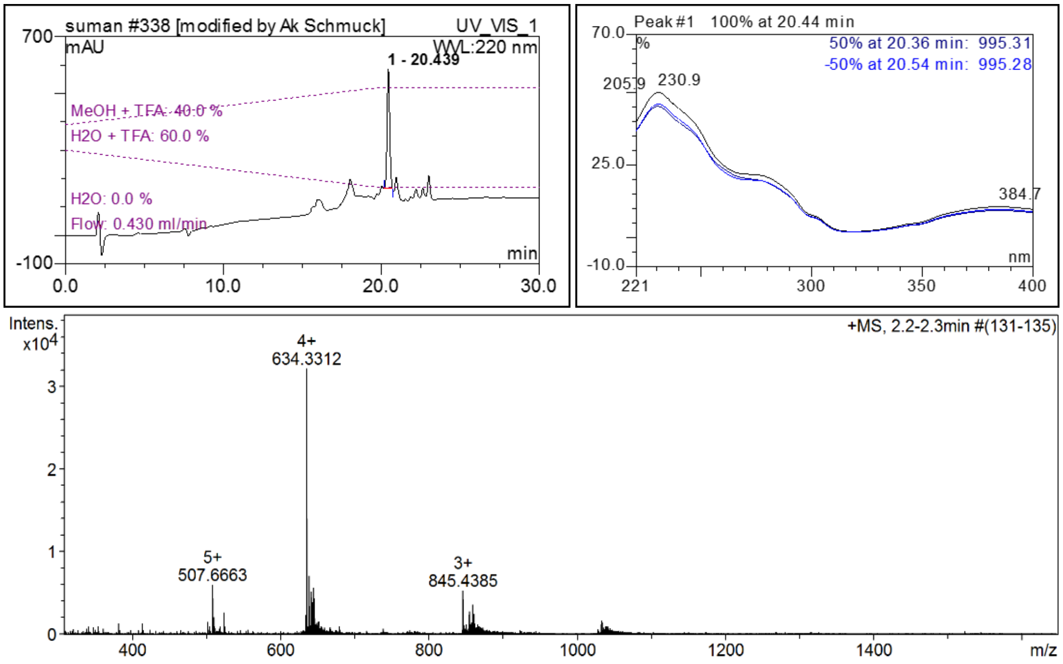

(A)

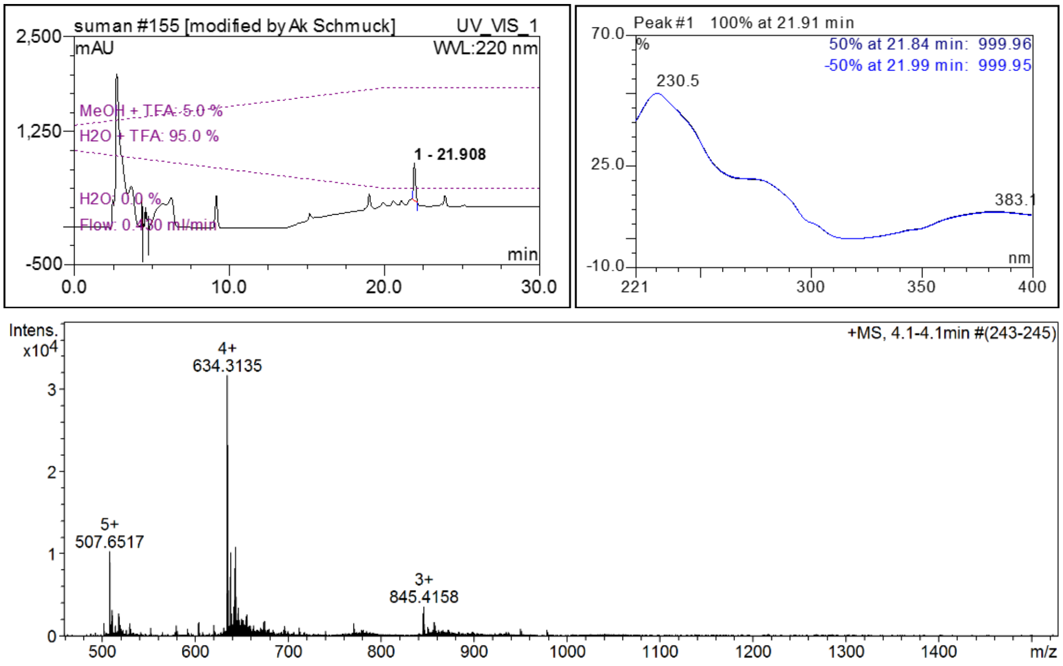

(B)

Figure S8: (A) Analytical HPLC and mass spectra of purified peak b from peptide **2a** gossypol conjugation reaction. (B) Analytical HPLC and mass analysis of purified peak III from peptide **2a** gossypol conjugation reaction.

Control Experiments for Structure Elucidation

In order to elucidate the structure of the active component in the mixture obtained from the reaction described on page S17, a number of control experiments on model systems were conducted in order to exclude formation of side products resulting from reactions between the Guanidinium moiety and Gossypol.

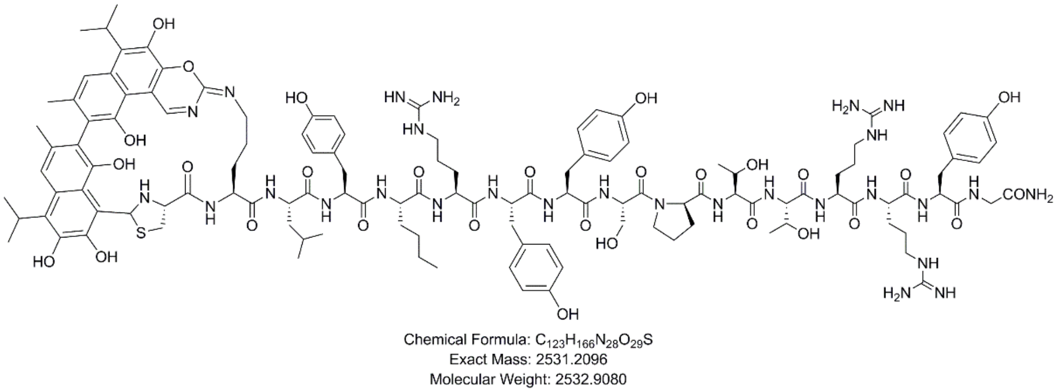

Figure S9: Structure of potential side products (not formed).

Ligation of Cys-RL with gossypol

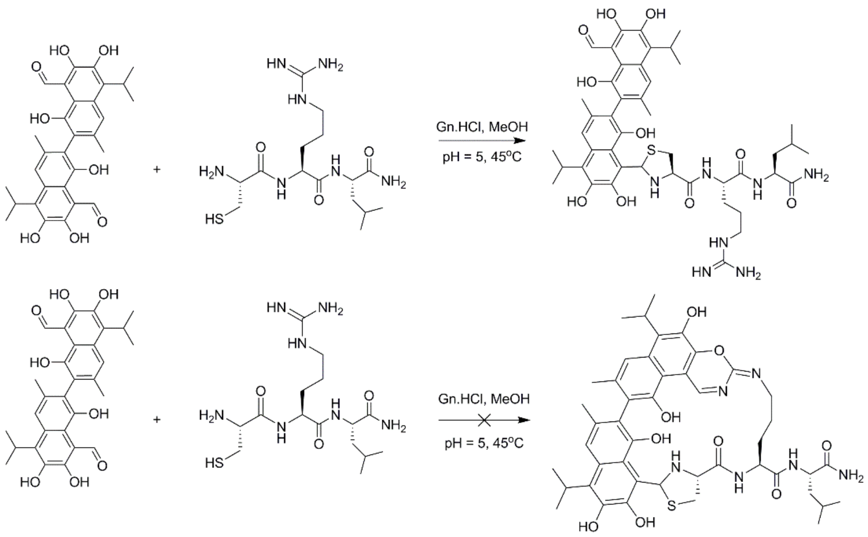

Model tripeptide Cys-RL was synthesized by Foc based SPPS. The HPLC analysis was carried out on a C18 analytical column using a gradient of 5-100% B over 20 min (t = 10.31 min). For preparative HPLC, 20-100% B over 60 min gradient was used to purify the peptide to the desired product in ~62% yield. **MS m/z (ESI$^+$):** 390.2 (M+1H$^+$, 100%), calc. 390.2

Cys-RL (3 mg, 7.7 × 10^{-3} mmol) was dissolved in 100 µL of 6 M Gn·HCl, 200 mM phosphate buffer at pH 5 in a reaction vial (Eppendorf). In another reaction vial, gossypol (6 mg, 11.5× 10^{-3} mmol) was dissolved in 500 µL methanol. The peptide solution was added to the gossypol solution in methanol and the reaction mixture was incubated at 45 ºC for 2h. Monitoring the progress of the reaction by analytical HPLC using C18 column with a gradient of 40-100% buffer B over 30 min revealed the appearance of peak a (t = 20.58 min) and peak b (t = 21.55 min). After completion of the reaction the reaction mixture was diluted with 500 µL MeOH : H_2O (1:1) solution and subjected to purification by preparative HPLC with a gradient 50-100% buffer B over 60 min. After collecting peak a and peak b, methanol was evaporated by rotary evaporator and the concentrated solution was lyophilized to afford the thiazolidine linked model peptide-gossypol conjugates (0.36 mg, 5.1% isolated yield for peak a; 0.42 mg, 6.1% isolated yield for peak b). **MS m/z (ESI^+):** Peak a: 890.4 ($M+1H^+$, 100%, calc. 890.4); **MS m/z (ESI^+):** Peak b: 890.4 ($M+1H^+$, 100%, calc. 890.4).

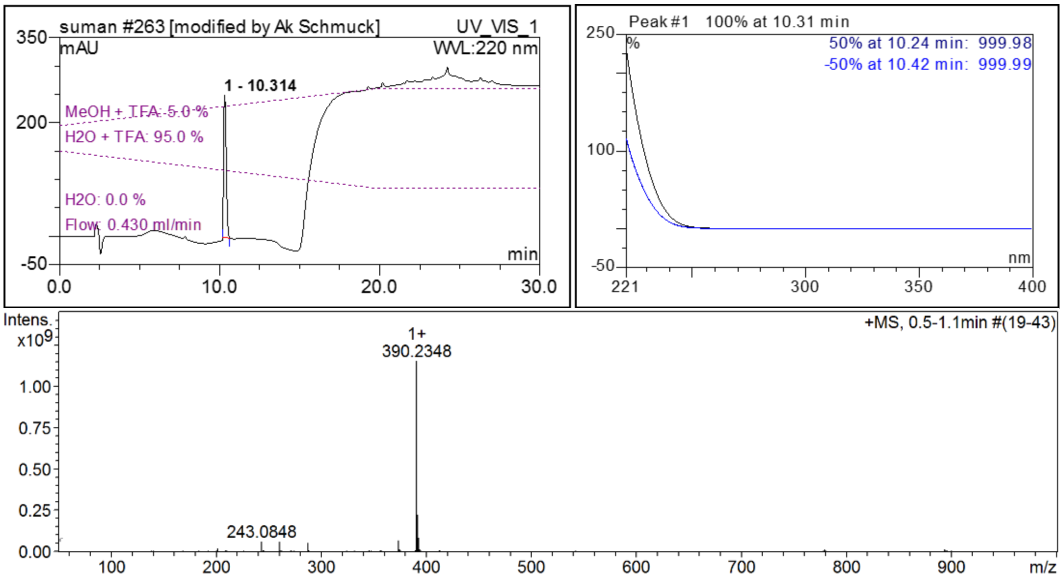

Figure S10A: Analytical HPLC and mass spectra of (A) purified CRL tripeptide with the observed mass 390.2 Da, calcd 390.2 Da.

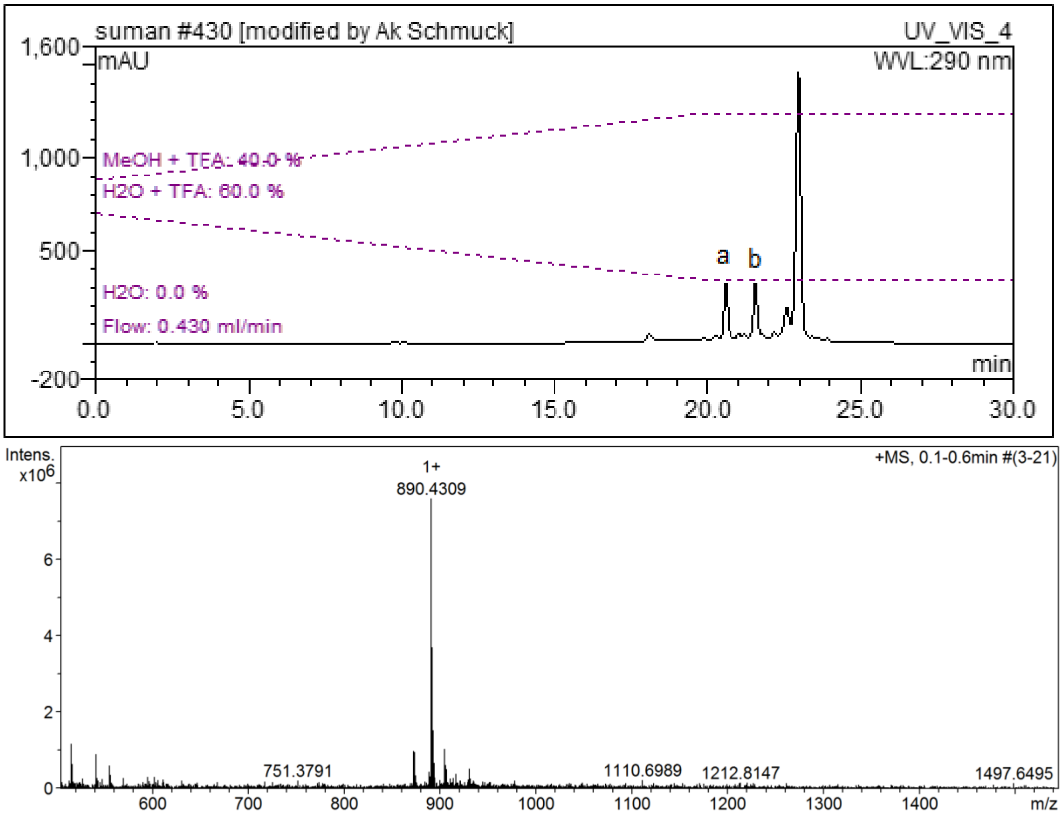

Figure S10B: Ligation reaction between CRL and gossypol and the mass of the purified product b with the observed mass 890.4 Da, calcd 890.4 Da.

Ligation of Fmoc-Arg-OH and gossypol

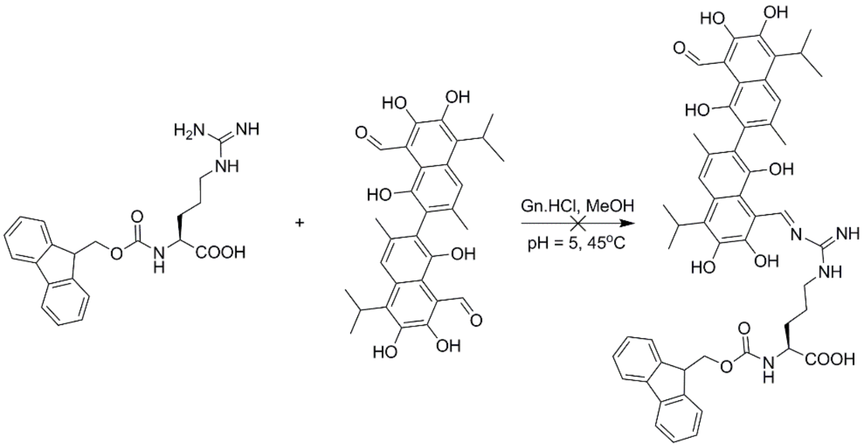

Fmoc-Arg-OH (3 mg, 7.5 × 10^{-3} mmol) was dissolved in 100 µL of 6 M Gn·HCl, 200 mM phosphate buffer at pH 5 in a reaction vial (Eppendorf). In another reaction vial, gossypol (6 mg, 11.5× 10^{-3} mmol) was dissolved in 500 µL methanol. The peptide solution was added to the

gossypol solution in methanol and the reaction mixture was incubated at 45 ºC for 3h. Progress of the reaction was monitored by analytical HPLC using C18 column with a gradient of 40-100% buffer B over 30 min. No product formation was observed and starting materials were recovered.

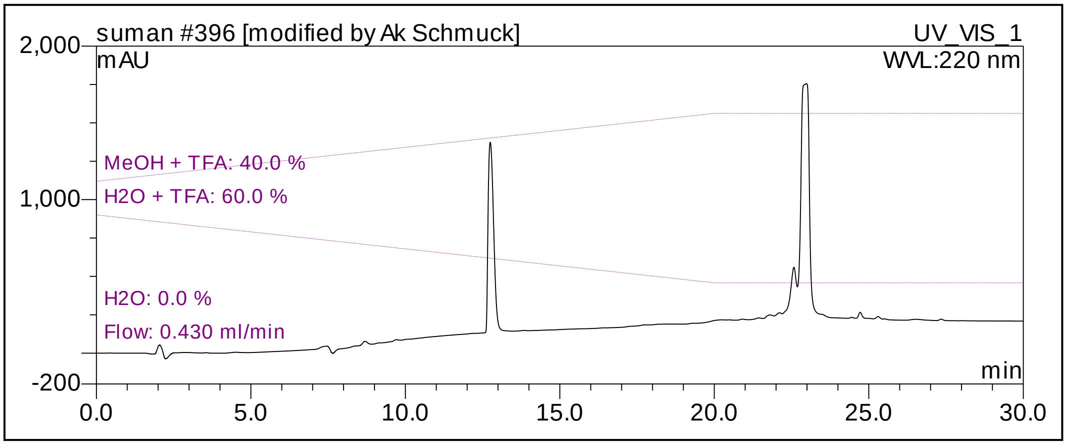

Figure S11: Analytical HPLC of ligation between Fmoc-Arg-OH and gossypol after 3h. First peak corresponds to Fmoc-Arg-OH and second peak corresponds to gossypol.

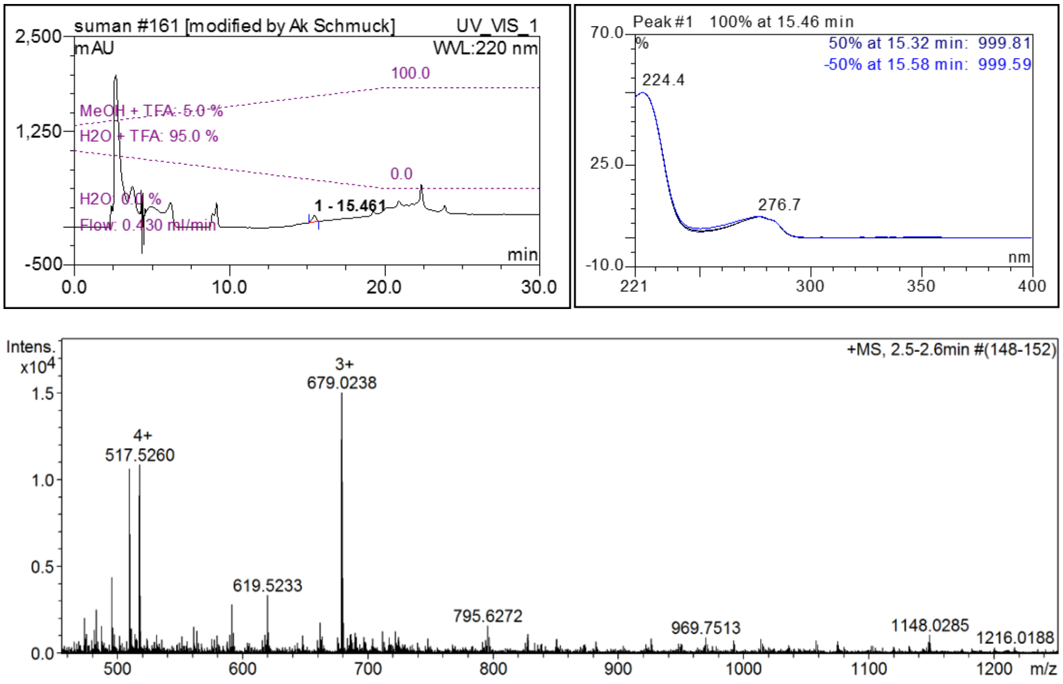

Figure S12: Analytical HPLC and mass spectra of desulfurized peptide released from the petide-**2a**-gossypol conjugate (peak b). **MS m/z (ESI$^+$):** 509.5 (M+4H$^+$, 61%), 517.5 (M+4H$^+$, 65%), 679.0 (M+3H$^+$, 100%).

Cell culture and cytotoxicity assay

Breast cancer MCF-7 cells and HeLa cells were cultured in Dulbecco's Modified Eagle's Medium (DMEM,Gibco), which was supplemented with 10 % FBS, streptomycin sulfate (0.1 mg ml^{-1}), penicillin (10 U ml^{-1}) and amphotericin B (0.25 µg ml^{-1}). Fibroblasts were cultured in RPMI medium (Gibco), which was supplemented with with 10 % FBS, streptomycin sulfate (0.1 mg ml^{-1}), penicillin (10 U ml^{-1}) and amphotericin B (0.25 µg ml^{-1}).

Cells were seeded (10^4 cells/well) on 96 well plates and co-incubated with peptide-drug conjugates in concentrations ranging from 2 to 150 µM for 12, 24 or 48 h in a humidified 5 % CO_2 atmosphere at 37 ºC. Cytotoxicity assay was performed by determining the number of remaining viable cells. The cell viability was measured via CellTiter 96® AQueous One Solution Cell Proliferation Assay (Promega) according to the manufacturer's instructions. After 1 h of incubation in a humidified 5 % CO_2 atmosphere at 37 ºC, absorbance was measured at 490 nm using a Glomax multi plate reader (Promega). Three replicates were generated per condition.

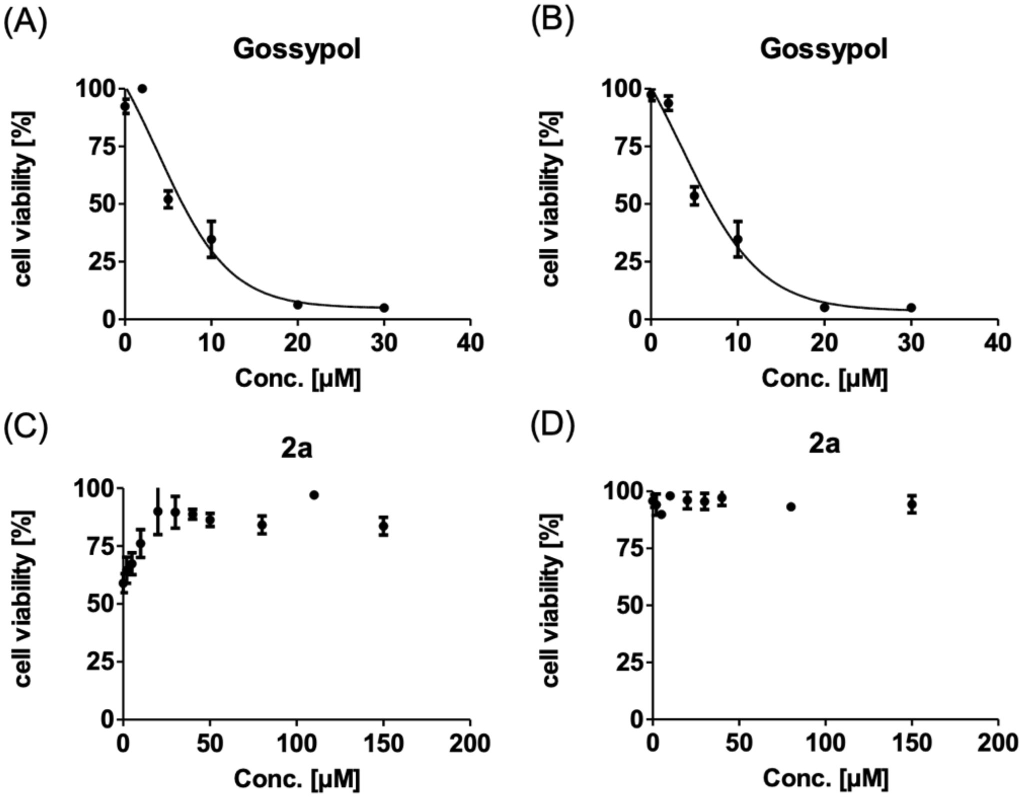

Figure S13: Cytotoxicity assay with Gossypol (A) on MCF-7 and (B) HeLa cells. Cytotoxicity assay with peptide **2a** on (C) MCF7 and (D) HeLa cells. MCF-7 and HeLa cells were treated with different concentrations of Gossypol or peptide 2a or the respective amounts of medium and incubated for 48 h before the number of viable cells was measured using the CellTiter 96® AQueous One Solution Cell Proliferation Assay (Promega). The absorbance (490 nm) values of the samples were substracted by the medium only control. Absorbance is depicted relative to the highest absorbance measured for each experiment, which was named 100% viability. Depicted are the average values obtained from the experiments carried out in triplicate. Error bars depicture the standard deviation.

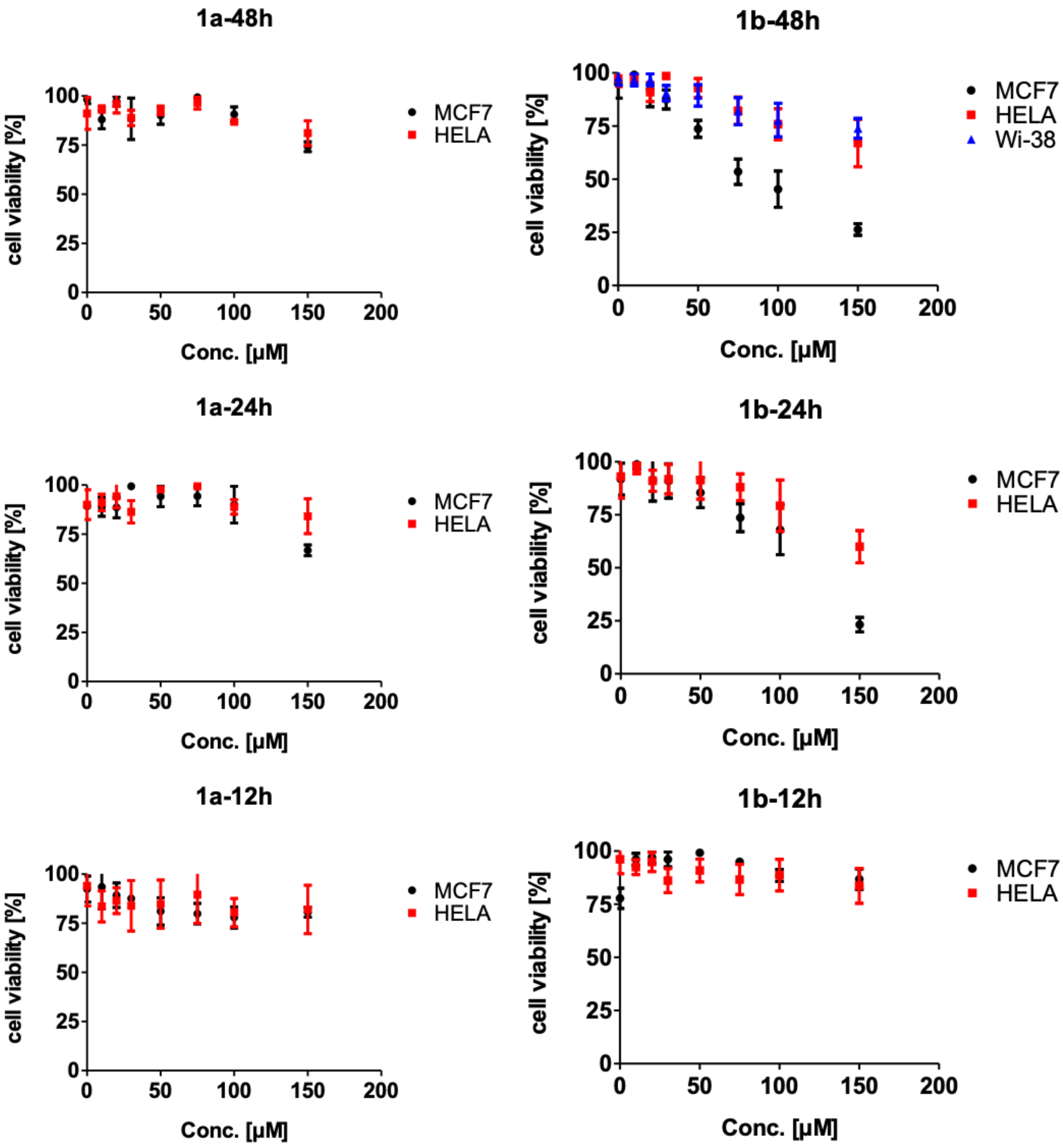

Figure S14: Toxic effects of **1a** and **1b** determined in a time-dependent cytotoxicity assay. MCF-7 and HeLa cells were treated for 12, 24 and 48 hours with different concentrations of the two differently linked peptide-drug conjugates **1a** and **1b**. WI-38 cells were incubated with the imine linked conjugate **1b** for 48h**.** Cell viability was measured using the CellTiter 96® AQueous One Solution Cell Proliferation Assay (Promega), the absorption values (490 nm) corresponding to each drug concentration were obtained by subtracting the medium only control from all data points and normalizing the absorption to the highest absorption measured in each experiment, which was named 100% viability. Depicted are the average values obtained from the experiments carried out in triplicate. Error bars depicture the standard deviation.

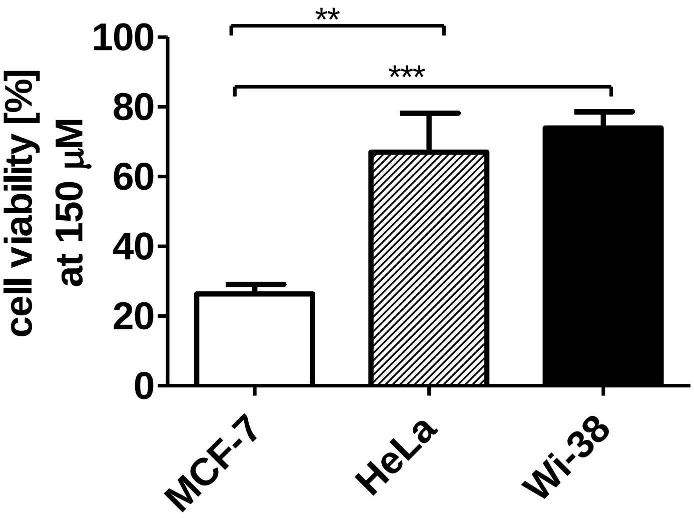

Figure S15: Comparison of toxic effects of **1b** on different cell types at 150 µM after 48 h.
**: P<0,01; ***: P<0,001

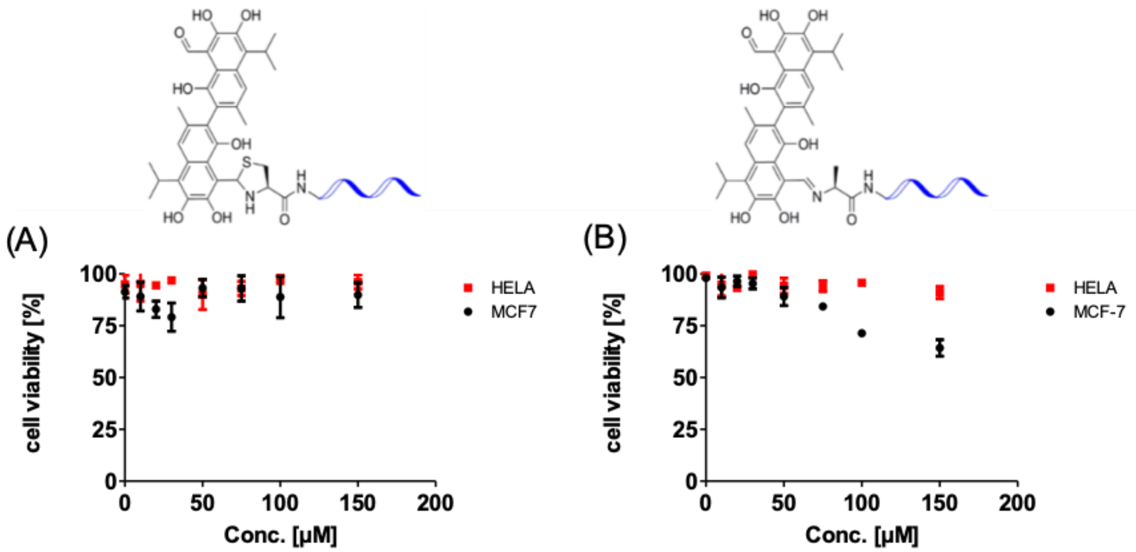

Figure S16: Cytotoxicity assay after 48h treatment with the diastereomers of (A) **1a** and (B) **1b** obtained from the first HPLC-peak. MCF-7 and HeLa cells were treated with different concentrations of the conjugates or the respective amounts of medium and incubated for 48 h before the number of viable cells was measured using the CellTiter 96® AQueous One Solution Cell Proliferation Assay (Promega). The absorbance (490 nm) values of the samples were substracted by the medium only control. Absorbance is depicted relative to the highest absorbance measured for each experiment, which was named 100% viability. Depicted are the average values obtained from the experiments carried out in triplicate. Error bars depicture the standard deviation.

Kinetics study of the cleavage of the imine and thiazolidine linkages

0.1 mg of the imine or thiazolidine linked peptide-drug conjugate was dissolved in 50 μL of 6M Gn·HCl, 200 mM phosphate buffer at pH 7 and incubated at 37 ºC. Analytical HPLC spectra were recorded (using C18 column with a gradient of 40-100% buffer B over 20 min and measuring the UV at 220 nm) at different time intervals taking 3 μL of the reaction mixture and diluting with 50 μL of methanol. The half-life of the imine linked peptide-drug conjugate was determined by integrating the peaks corresponding to the cleaved peptide and the intact peptide-drug conjugate, which revealed 52% cleavage of the peptide after 10 hours.

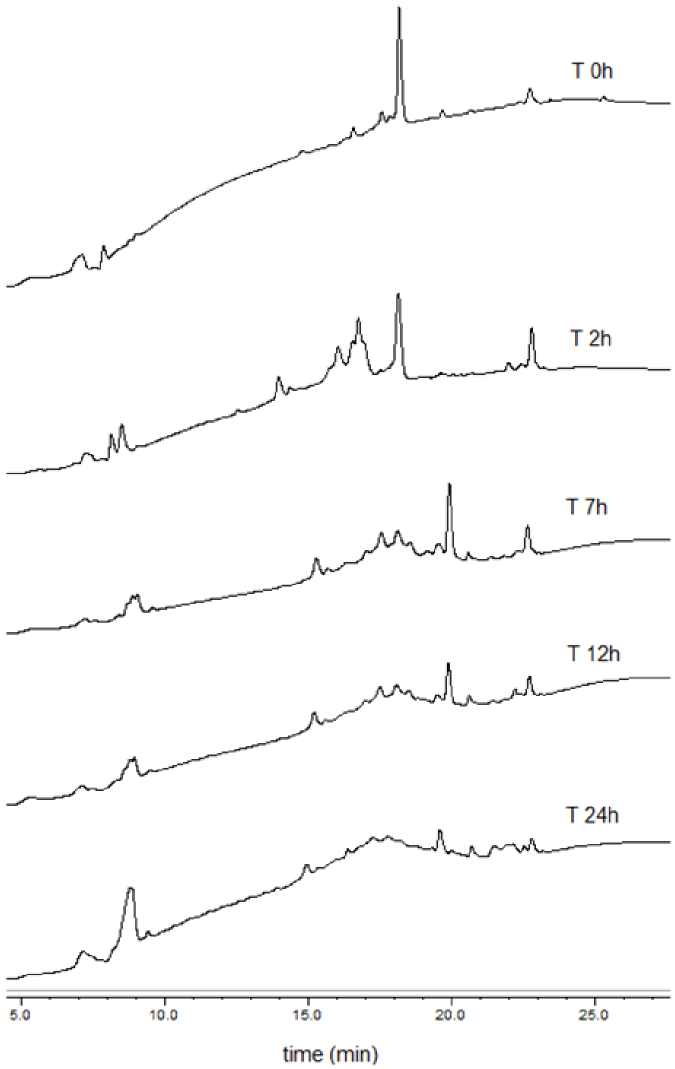

Figure S17A: Analytical HPLC studies of the cleavage kinetics of **1a**. Solvent gradient for HPLC was 40-100% B in 20 min; buffer B was MeOH with 0.1% TFA and UV was measured at 220 nm.

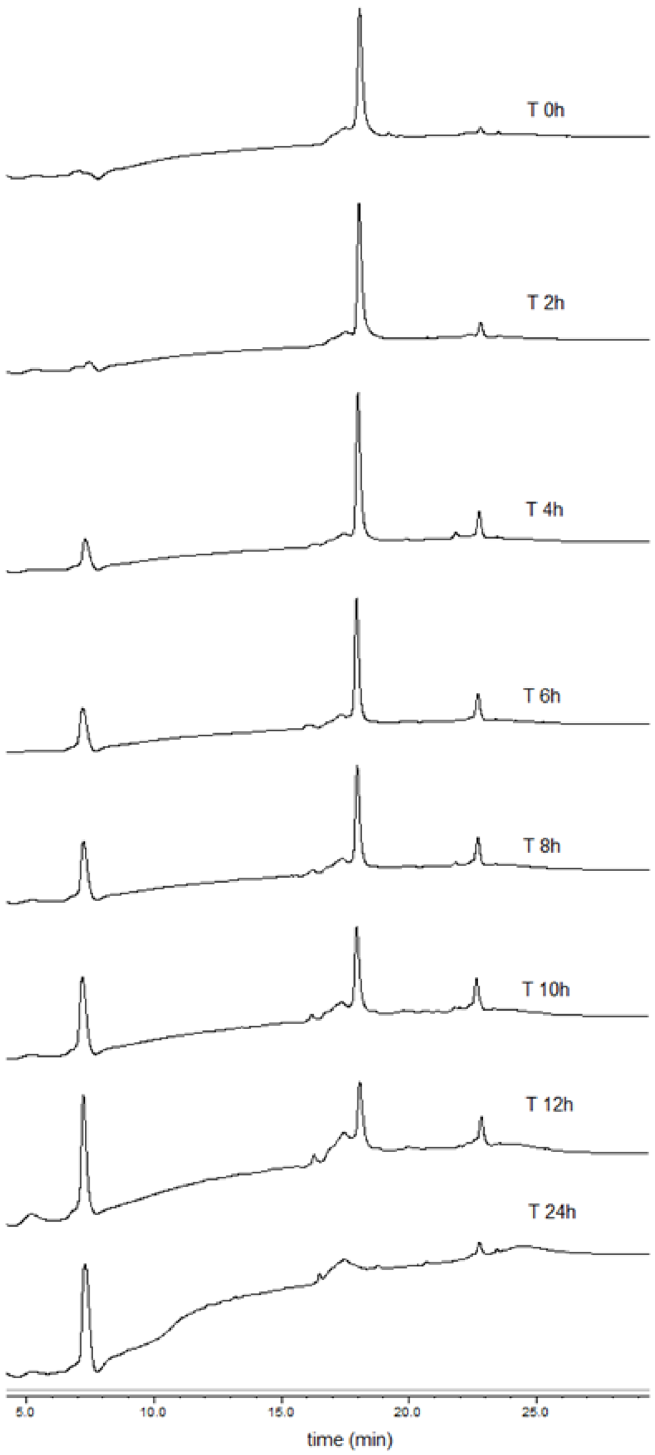

Figure S17B: Analytical HPLC studies of the cleavage kinetics of **1b**. Solvent gradient for HPLC was 40-100% B in 20 min; buffer B was MeOH with 0.1% TFA and UV was measured at 220 nm. Integration of the peaks corresponding to the cleaved peptide and the intact peptide-drug conjugate revealed that 52% of the conjugate was cleaved after 10 hours.

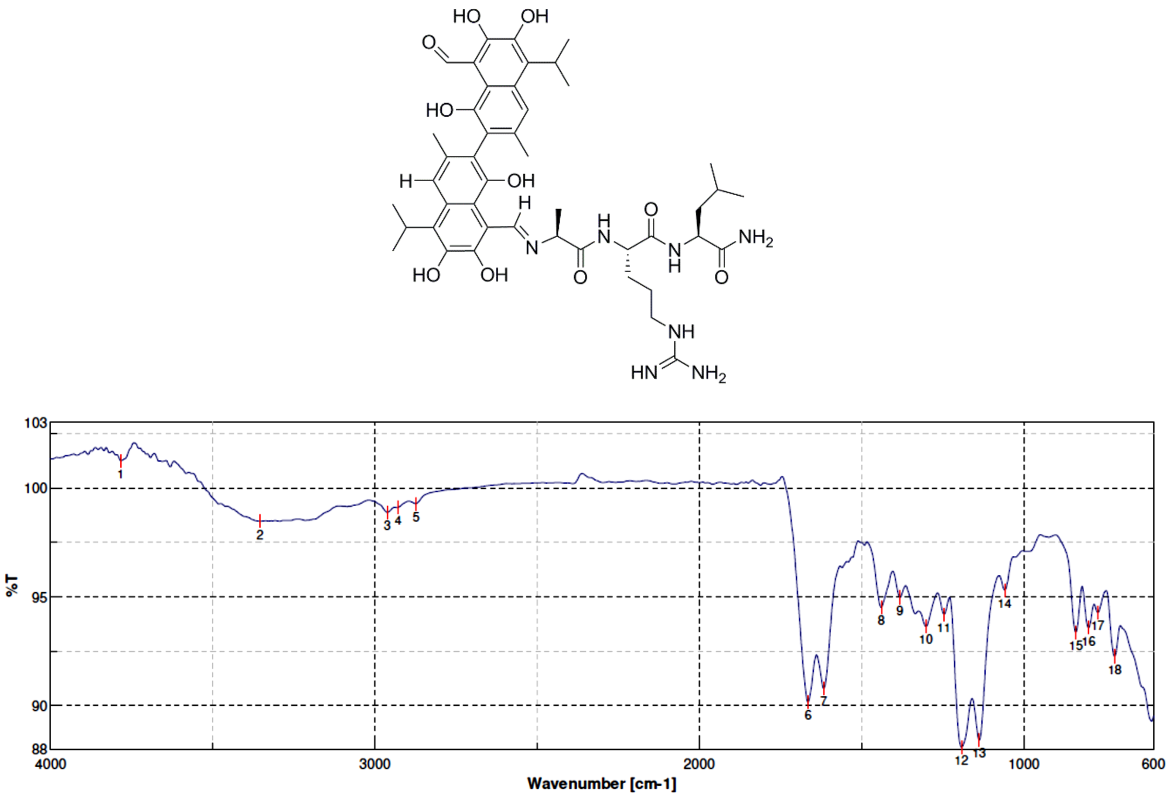

Figure S18: IR spectrum of the isolated product (peak b) of ligation between model tripeptide ARL and gossypol.
